# A MoClo-Compatible Toolbox of ECF Sigma Factor-Based Regulatory Switches for Proteobacterial Chassis

**DOI:** 10.34133/bdr.0025

**Published:** 2024-02-21

**Authors:** Doreen Meier, Christian Rauch, Marcel Wagner, Paul Klemm, Patrick Blumenkamp, Raphael Müller, Eric Ellenberger, Kinnari M. Karia, Stefano Vecchione, Javier Serrania, Marcus Lechner, Georg Fritz, Alexander Goesmann, Anke Becker

**Affiliations:** ^1^ Center for Synthetic Microbiology (SYNMIKRO) and Department of Biology, Philipps-Universität Marburg, Marburg, Germany.; ^2^ Bioinformatics and Systems Biology, Justus-Liebig-Universität Giessen, Giessen, Germany.; ^3^ The University of Western Australia, School of Molecular Sciences, Perth, Australia.

## Abstract

The construction of complex synthetic gene circuits with predetermined and reliable output depends on orthogonal regulatory parts that do not inadvertently interfere with the host machinery or with other circuit components. Previously, extracytoplasmic function sigma factors (ECFs), a diverse group of alternative sigma factors with distinct promoter specificities, were shown to have great potential as context-independent regulators, but so far, they have only been used in a few model species. Here, we show that the alphaproteobacterium *Sinorhizobium meliloti*, which has been proposed as a plant-associated bacterial chassis for synthetic biology, has a similar phylogenetic ECF acceptance range as the gammaproteobacterium *Escherichia coli*. A common set of orthogonal ECF-based regulators that can be used in both bacterial hosts was identified and used to create 2-step delay circuits. The genetic circuits were implemented in single copy in *E. coli* by chromosomal integration using an established method that utilizes bacteriophage integrases. In *S. meliloti*, we demonstrated the usability of single-copy pABC plasmids as equivalent carriers of the synthetic circuits. The circuits were either implemented on a single pABC or modularly distributed on 3 such plasmids. In addition, we provide a toolbox containing pABC plasmids compatible with the Golden Gate (MoClo) cloning standard and a library of basic parts that enable the construction of ECF-based circuits in *S. meliloti* and in *E. coli*. This work contributes to building a context-independent and species-overarching ECF-based toolbox for synthetic biology applications.

## Introduction

In synthetic biology, interchangeable biological parts are key to construct genetic circuits with predictable properties in the background of different host cells. Toward this goal, extensive studies have been conducted in recent years on orthogonal and tunable genetic switches. Apart from well-known transcription factors, various other types of transcriptional regulators have been engineered and analyzed in bacteria, especially in the model organism *Escherichia coli*. These regulators include small transcription-activating RNAs [1] variants of the T7 bacteriophage-derived RNA polymerase (RNAP) [2,3], or dCas9-based approaches [4,5]. Furthermore, synthetic regulatory switches based on alternative σ factors (σs) of the σ^70^ family were employed [6–8]. As dissociable subunits of the bacterial core RNAP, σs are important for promoter recognition and transcription initiation [9].

Extracytoplasmic function sigma factors (ECFs) constitute the most minimalistic and diverse group of alternative σs with only 2 (σ_2_ and σ_4_) out of 4 conserved domains found in housekeeping σs. ECFs are typically stress-induced and recruit the RNAP to a subset of promoters through selective recognition of the −10 and −35 promoter motifs mediated by the σ_2_ and σ_4_ domains, respectively [10]. Due to the lack of σ_2_ region 1.2 and σ_3_ other promoter elements such as the discriminator and the extended −10 motif cannot be recognized by ECFs [11]. On average, 10 ECF-encoding genes are found in bacterial genomes, and comparative computational analyses identified more than 150 phylogenetically distinct ECF groups [12]. It was further suggested that members of a phylogenetic group recognize promoters with similar core motifs, whereas they are unable to activate promoters associated with ECFs from other groups [12,13]. Indeed, in a comprehensive pilot study, Rhodius et al. [8] identified 20 heterologous ECFs from different phylogenetic groups that activate their cognate promoters in the γ-protobacterium *E. coli* in a highly orthogonal manner. This high specificity of ECFs for their cognate promoters is reflected in a recent computational mutual information analysis that predicted conserved interactions between amino acid residues in key positions of ECFs and nucleotides of their cognate promoters [14]. Accordingly, ECF promoter specificities derived from this information were confirmed in vivo, and ECF regulons were predicted [14].

Functionality of ECFs from different bacterial classes in *E. coli* [8] can be explained by sufficient conservation of RNAP subunits [15,16]. However, this is most likely not the case in all bacterial hosts. Attempts to implement ECF/promoter pairs as heterologous genetic switches in *Bacillus subtilis* revealed a rather narrow phylogenetic acceptance range, with a preference for those ECFs that were derived from Firmicutes and thus from members of the same phylum [17]. This raises the question of whether functional ECF/promoter pairs (ECF switches) need to be identified individually for each bacterial host or whether regulatory switches from established libraries can at least be transferred successfully between species of the same phylum, assuming a similar acceptance range even in distantly related bacteria.

It was shown that ECFs are suitable building blocks to generate multistep genetic circuits. Two or more ECFs were combined in cascades resulting in delayed activation of a reporter gene in *E. coli* and *B. subtilis* [7]. The behavior of these regulatory cascades was comparable, although different sets of ECFs were used as regulators in these 2 bacterial species [7]. Functionality of these delay circuits was shown in multiple copies using a plasmid-based approach or in single copy by chromosomal integration [7,18]. The use of genetic circuits in single copy could be advantageous to conserve cellular resources for transcription and translation and reduce the burden on the host cell, especially as circuits increase in size and complexity. For example, it has experimentally and theoretically been shown that the functionality of synthetic gene circuits is coupled to the limited amount of RNAP and ribosomes in a cell [19–21].

In this study, we identified a common set of ECF-based regulators for dual use in the γ-proteobacterium *E. coli* and the α-proteobacterium *Sinorhizobium meliloti*. The latter is a nitrogen-fixing plant symbiont, which was proposed as a candidate rhizobial chassis for targeting plant roots and designing new symbiotic systems by de Lorenzo et al. [22]. Besides, in its free-living state, *S. meliloti* is also an emerging host for the biotechnological production of natural compounds [23,24]. We show that genetic switches and 2-step delay circuits composed of the same set of ECF/promoter pairs and implemented in single copy in *E. coli* and *S. meliloti* exhibit similar properties. This was the case despite of different implementation strategies—chromosomal integration in *E. coli* and introduction on single-copy plasmids in *S. meliloti*. The latter strategy facilitates modular assembly of genetic circuits. Furthermore, we expand the toolkit of basic parts recently published for construction of ECF-based regulatory circuits in *E. coli* using the Golden Gate modular cloning (MoClo) standard [25] by new parts and plasmids for *S. meliloti* and most likely for closely related α-proteobacteria.

## Materials and Methods

### Bacterial strains and growth conditions

*E. coli* strains used in this study are listed in Table S1. Strains were grown at 37 °C in Lysogeny Broth (LB) liquid medium with aeration (shaking at 250 rpm) or on 1.5% LB agar plates. Alternatively, Mops minimal medium (Teknova catalog no.: M2106, 0.5% glycerol as carbon source) was used for cultivation. Media were supplemented with the following antibiotics to select for cells with desired plasmids or for plasmid maintenance: chloramphenicol (25 μg/ml), kanamycin (50 μg/ml), gentamicin (6 μg/ml), spectinomycin (100 μg/ml), and hygromycin B (50 μg/ml). For selection and growth of strains with gene circuits integrated into the chromosome, a chloramphenicol concentration of 6 μg/ml was used. *S. meliloti* strains used in this study are listed in Table S1. They were grown at 30 °C with aeration or shaking at 200 rpm either in complex tryptone (TY) medium [26] or in modified Mops-buffered minimal medium [27] (10 g/l Mops, 10 g/l mannitol, 1 g/l NH_4_Cl, 0.1 g/l NaCl, 0.246 g/l MgSO_4,_ 250 mM CaCl_2_, 10 mg/l FeCl_3_•6H_2_O, 3 mg/l H_3_BO_3_, 2.23 mg/l MnSO_4_•4H_2_O, 1 mg/l biotin, 0.3 mg/l ZnSO_4_•7H_2_O, 0.12 mg/ml NaMoO_4_•2H_2_O, 0.065 mg/ml CoCl_2_•6H_2_O, and 2 mM K_2_HPO_4_). For solid TY agar plates, 1.5% (w/v) agar was added. When appropriate, solid and liquid media were supplemented with the following antibiotics: gentamicin (30 and 10 μg/ml), spectinomycin (200 and 100 μg/ml), kanamycin (200 and 100 μg/ml), hygromycin (50 and 50 μg/ml), and streptomycin (600 and 300 μg/ml).

### DNA manipulation and extraction methods

Plasmids were constructed using various DNA assembly methods. Enzymes were purchased from Thermo Fisher Scientific unless otherwise stated. Besides standard molecular cloning techniques [28], the MoClo DNA assembly method was used [25]. Restriction/ligation reactions were set up in a final volume of 20 μl using 15 fmol of each DNA part (plasmid or polymerase chain reaction [PCR] product), 1 μl of the required type IIS restriction enzyme (either BpiI or BsaI), 1 μl of T4 DNA ligase (5 U/μl), and 2 μl of T4 DNA ligase buffer. Reactions were incubated for 5 h at 37 °C and then heat-inactivated for 10 min at 50 °C and 10 min at 80 °C. Alternatively, the Gibson method, using the 1-step isothermal DNA assembly protocol [29] and the Aqua cloning technique [30], were used for plasmid assemblies. The ligase chain reaction method [31] was used to build MoClo-compatible pABC plasmids as described by Döhlemann et al. [32]. PCRs were performed with Q5 polymerase (New England Biolabs [NEB]; Ipswich, MA, USA) or with Taq polymerase (NEB; Ipswich, MA, USA). Primers were purchased from Integrated DNA Technologies (Leuven, Belgium). Sequences can be extracted from Table S2. Genomic DNA, plasmid DNA, or synthesized double-stranded DNA fragments (Integrated DNA Technologies) were used as template. Sequences from synthesized double-stranded DNA fragments can be found in Table S2. Genomic DNA was isolated using the DNeasy Blood & Tissue Kit (Qiagen; Venlo, Netherlands). Plasmid DNA was purified using the E.Z.N.A. Plasmid Mini Kit (Omega Bio-Tek; Norcross, GA, USA). DNA 5′ DNA ends were phosphorylated by T4 Polynucleotide Kinase (Thermo Fisher Scientific; Waltham, MA, USA). 5′ DNA ends were dephosphorylated by FastAP Thermosensitive Alkaline Phosphatase. DNA-reaction mixtures were purified using the E.Z.N.A. Cycle-Pure Kit (Omega Bio^-^Tek). The illustra GFX PCR DNA and Gel Band Purification Kit (GE Healthcare Life Sciences; Chalfont St Giles, GB) was used for gel extraction of DNA. Correct assembly of plasmids was verified by sequencing (Eurofins Genomics; Köln, Germany). A more detailed description of plasmid cloning strategies can be found in Table S3.

#### MoClo standard: Construction of gene circuits for characterization in *S. meliloti* and *E. coli*

We utilized a subset of MoClo vectors that were originally devised for the eukaryotic science community [25] or compatible plasmids generated in this or in previous studies [7,18] for the standardized assembly of gene circuits. DNA assemblies rely on Golden Gate cloning with type IIs endonucleases. In some cases, subcloning of assembled transcription units or multigene circuits into plasmids that are noncompatible with the MoClo assembly strategy was required.

#### MoClo-standards and parts

According to the original MoClo syntax, basic parts, which are promoters, ribosome binding sites (RBS), coding sequences, and terminators in our case, are carried by level 0 vectors with a spectinomycin resistance gene [25]. All basic parts are flanked by BsaI sites in opposing orientation and unique 4-bp fusion sites. BsaI is used together with DNA ligase in a single cloning step to assemble transcriptional units from selected level 0 parts into level 1 destination vectors conferring ampicillin resistance [25]. Since transcriptional units are flanked by opposing BpiI sites, they can be further assembled into multigene constructs using level M or level 2 destination vectors whose selection markers differ from level 1 plasmids [25,33]. *Ecf* circuits for *E. coli* were recently created using the MoClo standard [7]. We adopted the specific organization of basic parts in level 0 vectors and expanded the number of available level 0 *ecf* and P*_ecf_* plasmids (Fig. S1 and Table S3). Besides, we generated several level 0 vectors carrying inducible or constitutive promoters, RBS, and terminators suitable for *S. meliloti* that were characterized in more detail (Fig. S1). If necessary, basic parts were cleared for BpiI and BsaI sites.

#### Assembly of gene circuits for chromosomal integration into *E. coli*

For cloning of synthetic gene circuits and subsequent characterization in *E. coli*, we utilized MoClo-compatible CRIMoClo vectors in the final cloning step. These allow for plasmid integration into 1 of 4 orthogonal phage attachment sites (*attB*) in the *E. coli* genome [18].

#### Assembly of gene circuits for characterization in *S. meliloti*

Gene circuits composed of single transcription units for the characterization in *S. meliloti* were preassembled from basic parts into slightly modified MoClo level 1 destination vectors. They differ from those that are available in the original toolkit by additional type II restriction sites flanking the assembled DNA fragment. This allows subcloning of transcription units into the pABC plasmid series or into a broad-host-range pSRKGm [34] derivative (pSRKGm [m]) generated in this study. To allow MoClo assemblies of transcription units and multigene circuits directly into pABC vectors, we cleared the plasmids for BsaI and BpiI restriction recognition sites and carefully characterized them in terms of segregation stability and copy number in *S. meliloti* Rm1021 as detailed in the results. Basic parts (constitutive promoters, inducible promoters, and RBS) were characterized using a MoClo-compatible pABC Level 1 vector (Fig. S1) and a luminescence reporter. Different Rho-independent terminators were evaluated employing a MoClo-compatible Level M derivative of pSRKGm (pSRGm-M-1) and a dual-fluorescence reporter assay as detailed in Fig. S1.

### DNA transfer and strain development

Chemically competent *E. coli* strains were prepared according to the Inoue method [35]. Different chemically competent *E. coli* strains were transformed with plasmids or plasmid-assembly reactions by heat shock according to a standard protocol [28]. Plasmid integration of Level M compatible CRIMoClo vectors pSV004 or pSV006 carrying different gene circuits was performed as described elsewhere [18]. In particular, chemically competent *E. coli* SV01 strains carrying the CRIM helper plasmid pAH69 (in case of pSV004 derivatives) or the CRIM helper plasmid pAH121 (in case of pSV006 derivatives) were transformed with plasmid DNA. The helper plasmids encode a cognate recombinase mediating site-specific recombination at the HK022 or the P21 phage attachment site. After the regeneration step (incubation of heat-shocked cells in LB at 37 °C for 1 h), cells were kept at 42 °C for another hour to induce recombinase gene expression and to cure the helper plasmid. The cells were spread onto selective agar medium plates and incubated at 37 °C overnight. Conjugative matings were performed to transfer up to 3 *oriT* bearing pABC plasmids in parallel into *S. meliloti* [36]. Equal amounts of cells from *E. coli* S17-1 donor and *S. meliloti* recipient strains were mixed, spotted onto TY agar plates, and incubated at 30 °C overnight. Cell mixtures were then plated on selective media. After incubation at 30 °C for 3 d, visible colonies appeared. Alternatively, electrocompetent cells were prepared and transformed as described elsewhere [37].

### Microplate reader assays

*E. coli*: Microplate reader assays in *E. coli* were performed as described elsewhere [7]. Briefly, single colonies of cells with chromosomally integrated gene circuits were used to inoculate liquid LB medium (3 ml) with appropriate antibiotics. Stationary-phase cultures were diluted 1:6,000 into Mops minimal medium (3 ml) supplemented with appropriate antibiotics. Cultures were grown overnight to an optical density at 600 nm (OD_600_) of 0.5 to 0.6. Cultures were adjusted to an OD_600_ of 0.05, and 100 μl were transferred in wells of a microtiter plate (GREINER catalog no.: 655097). The plate was incubated in a plate reader at 37 °C without lid or membrane (Tecan Infinite F200 pro) with shaking at 3.5-mm amplitude. OD_600_ as well as luminescence were determined every 5 min. After 2 h, cells were induced with arabinose before incubation, and measurements were continued.

*S. meliloti*: Microplate reader assays in *S. meliloti* were performed as follows: Small amounts of single colonies were picked from selective agar plates and grown in 100 μl of TY using 96-well microtiter plates (GREINER catalog no.: 655161) sealed with a membrane (GREINER catalog no.: 676001). The next day, stationary cultures were diluted 1:500 into 150 μl of Mops minimal medium supplemented with antibiotics. The plate was again sealed with a membrane (GREINER catalog no.: 676001) and incubated overnight at 30 °C with shaking at 1,200 rpm, until the cultures reached an OD_600_ of ~0.5. An automated liquid handling platform (Tecan Freedom EVO 200) accommodating a plate reader (Tecan infinite M200), an incubator (LiCONiC StoreX STX220), and a shaker (Thermo Scientific TM Teleshake, 2-mm amplitude) was used for cell synchronization, incubation, induction of gene expression, and continuous measurement of luminescence and OD_600_. Briefly, the starting OD_600_ of cultures grown in individual wells of one or more microtiter plates was determined and adjusted to an OD_600_ of 0.05 using a black microtiter plate (GREINER catalog no.: 655097). The plates were covered with plastic lids featuring condensations rings (GREINER catalog no.: 656171). Before plate reader measurements or to add inducer, the lids could easily be removed by the liquid handling platform. After 3 h of incubation (30 °C, humidity of 80%) during which OD_600_ and luminescence were measured every ~25 min, isopropyl β-d-1-thiogalactopyranoside (IPTG) was added if required at indicated concentrations. Then, OD_600_ and luminescence were measured every ~20 min. Before each measurement cycle, plates were shaken at 1,250 rpm for 30 s. If detailed dose-response curves were not required, measurements were made at 3, 6, 9, and 12 h after induction of gene expression. When determining termination efficiencies based on a dual-fluorescence reporter assay similar as described in [38], OD_600_ as well as mCherry (extinction: 552 nm, emission: 612 nm) and mVenus (extinction: 515 nm, emission: 548 nm) signals were determined 3, 6, 9, and 12 h after cell synchronization.

### RNA purification and RNA-seq

*S. meliloti* strains were generated that carried either the empty vector pSRKGm [m] or the same plasmid backbone including a transcription unit for *ecf02* (pSRKGm [m] P_*lac*T5_ *ecf02*) or *ecf11* (pSRKGm [m] P_*lac*T5_ *ecf11*) expression. For sample collection, *S. meliloti* strains were grown to an OD_600_ of 0.1 to 0.3 in Mops buffered minimal medium with gentamicin (6 μg/ml). The OD_600_ was adjusted to 0.1 and IPTG was added at a final concentration of 500 μM. Cells were grown for ~ 8 to 9 h until cell density reached an OD_600_ of 0.8 and collected by centrifugation at 10,000 rcf for 5 min. Cell pellets were frozen in liquid nitrogen and kept at −80 °C. Assays were performed in triplicate for each strain. For total RNA isolation, the Qiagen miRNeasy Kit was used starting from a pellet of 8-ml cultured cells. The cell pellet was resuspended in 1 ml of QIAzol lysis reagent and homogenized in a FastPrep sample preparation system using Lysing Matrix B containing 0.1-mm silica beads (MP biomedicals) and the following settings: 3 × 6,500 rpm for 20-s, 15-s break. The supernatant was transferred into a new tube and RNA isolation was performed according to the manufacturer’s instruction including the optional on column deoxyribonuclease I digestion. RNA integrity was analyzed with an Agilent 2100 Bioanalyzer system using the Agilent RNA 6000 Nano Kit. Ribosomal RNA was depleted using the Illumina Ribo-Zero rRNA removal Kit (Bacteria). Complementary DNA libraries were prepared with the NEB Ultra RNA directional prep kit for Illumina followed by sequencing on an Illumina HiSeq 3000 system with 1 × 150-bp single reads. The reverse complement of each read was generated with the FASTX-Toolkit as the sequenced reads were complementary to the annotation. Sequencing reads were aligned using Bowtie2 v2.4.4 [39] to the reference genome. The Rm1021 genome sequence (chromosome: NC_020528.1, pSymA: NC_020527.1, pSymB: NC_020560.1) and genome annotation were taken from https://www.cebitec.uni-bielefeld.de/CeBiTec/rhizogate/ [40,41]. Sequencing reads were also mapped to the plasmids pSRKGm [m], pSRKGm [m] P_*lac*T5_ *ecf02*, and pSRKGm [m] P_*lac*T5_ *ecf11* if present. The mapped reads were assigned to “gene” features using featureCounts (subread v2.0.1) with strand-specific read counting ‘-s 1’ [42]. Differential gene expression analysis was done with Curare 0.3.1 (https://github.com/pblumenkamp/Curare). Briefly, normalization of read counts and comparison of gene expression between samples (Table S4) were done with DESeq2 1.34.0 [43]. Sequencing data are available from ArrayExpress (reference number: E-MTAB-12126).

### Cappable-seq

Total RNA from the same samples that were subjected to RNA sequencing (RNA-seq) were also used for Cappable-seq [44]. However, RNAs from triplicates were pooled in equal amounts before library preparation. Cappable-seq was performed by Vertis Biotechnologie AG in Freising, Germany (https://www.vertis-biotech.com/home). Briefly, 5′-triphosphorylated RNAs were capped with a 3′-desthiobiotin-TEG-guanosine 5′-triphosphate (NEB) using the vaccinia capping enzyme (NEB). This allows capturing of biotinylated RNA species on streptavidin beads and the elution of enriched 5′-fragments of primary transcripts [44]. To discriminate 5′-triphosphorylated RNAs from contaminating processed or degraded RNA molecules with a 5′-monophosphate, they were differentially labeled with identifying sequence tags (TSS-tag and PSS-tag) during RNA library preparation. The generated complementary DNA pool was sequenced on an Illumina NextSeq 500 system using 75-bp read length. Sequencing data are available from ArrayExpress (reference number: E-MTAB-12127). Cappable-seq reads were mapped with Bowtie2 v2.4.4 using '--all', '--mm', and '--very-sensitive' settings [39], and all nonbest mappings of single reads were filtered. TSS detection was performed according to [44] with modifications as specified in [45]. Briefly, the first base of a TSS read determines the TSS position and was used to build ‘alignments per base’ scores (Rns). Relative read scores (RRS) were calculated for each TSS using a slightly modified formula from [44]: RRS = (Rns/Rt) × 10^6^, where Rt represents the total number of mapped TSS reads. A local accumulation of several TSS was defined as TSS cluster if the gap between 2 neighboring TSS did not exceed 3 nt. The position of the dominant TSS within the cluster was used as reference. TSS with a minimal RRS of 1.5, 3.0, and 7.5 were extracted (Table S5). For subsequent analysis, TSS with a minimal RRS of 3.0 were considered.

### Prediction of ECF02 and ECF11 promoter motifs upstream of strain specific TSS

ECF02- and ECF11-specific TSS on the chromosome, pSymA and pSymB as compared to the control strain were extracted (Table S6). Upstream sequences (40 nt, including the TSS) of ECF02- and ECF11-specific TSS were analyzed for the presence of promoter motifs overlapping with ECF02- and ECF11-specific promoter preferences (Table S6). Promoter preferences are represented by position-specific scoring matrices (PSSMs) that were retrieved from Todor et al. [14] and are shown in Table S6. The best match for the −35 and −10 motif of ECF02 and ECF11 with a spacer distance of 15 to 17 nt and a distance of 3 to 6 nt between the end of the −10 motif and the TSS were determined based on the respective log-likelihood PSSMs using a Perl script (available on https://gitlab.uni-marburg.de/synmikro/ag-lechner/ecf-offtarget-prediction). The total promoter score is determined by addition of the −35 and −10 log motif scores. Negative promoter scores closer to 0 have greater similarities to the promoter PSSM. *E*-values were calculated based on 1,000 random sequences of the same mononucleotide distribution as the retrieved region (probability of observing scores at least as extreme as the actually obtained score). Promoters with *E*-values below or equal to 0.05 were retained (Table S6).

### Computational prediction of off-target transcription from the host genome

To predict promoters recognized by heterologous ECFs that may lead to the overexpression of genes from the Rm1021 genome, sequences 500 nt upstream to 100 nt downstream of all annotated features were extracted and computationally analyzed. Sequences were scanned for *ecf* promoters and total promoter scores, and *E-*values were determined as described in the paragraph: Prediction of ECF02 and ECF11 promoter motifs upstream of strain-specific TSS. The same approach was applied to the whole Rm1021 genome to analyze the background distribution of putative *ecf* promoters. To set reasonable cutoffs for promoter predictions, we examined sequences upstream of operons or monocistronic units that were known to be up-regulated by *ecf* overexpression in an RNA-seq experiment (log_2_(*fold change*) fold change > 2, adjusted *P* value < 5%). For each regulated operon or monocistronic unit the Pareto optimal PSSM hits according to the −35 and the −10 motif score were identified. From these, we selected the worst values for the −35 motif and the −10 motif scores, the total promoter score, and the E-value as minimal cutoffs. To combat overfitting, minimal cutoff values were relaxed by an uncertainty factor of 5%. We applied these cutoffs to all *ecf* promoter-variants that were identified in the 500 nt upstream to 100 nt downstream region of annotated genes. The PromPredict tool with default parameters was used to further improve predictions, based on the observation that functional promoters are characterized by lower DNA duplex stability compared to flanking regions [46].

### Plasmid stability assays

To assess inheritance stability of MoClo-pABC plasmids with BsaI/BpiI cleared *repABC* regions in *S. meliloti* Rm1021, 3 strains carrying different sets of compatible MoClo-pABC vectors or derivatives were generated. Strain DM_Sm109_ carried pABCa-1-1a, pABCb-mod-1-1a-P*_min_*-*egfp*, and pABCc-1-1a-wo *lacZα*. In strain DM_Sm107_, plasmids pABCb-mod-1-1a, pABCa-1-1a-wo *lacZα*, and pABCc-1-1a-P*_min_*-*egfp* were introduced. Strain DM_Sm108_ harbored plasmids pABCc-1-1a, pABCa-1-1a-P*_min_*-*egfp* and pABCb-mod-1-1a-wo *lacZα*. *S. meliloti* cultures were grown over 5 d with 200-rpm shaking at 30 °C in nonselective TY medium supplemented with streptomycin. Cultures were diluted every day to maintain exponential growth (OD_600_ = 0.05 to 1) and dilution series were plated on nonselective TY agar plates with streptomycin. From the emerging colonies, 96 were examined for antibiotic resistance against gentamicin, hygromycin B and kanamycin, indicative of the presence of pABCa-1-1a, pABCb-mod-1-1a, and pABCc-1-1a or derivatives. Single colonies were resuspended in 0.9% NaCl and 5 μl of the suspension were spotted on agar plates with streptomycin, gentamicin, hygromycin B, and kanamycin, respectively. Growth indicated the presence of the assessed plasmid.

### Plasmid copy number determination

Plasmid copy number in *S. meliloti* was determined by quantitative PCR (qPCR) as previously described [32] with few modifications. Briefly, *S. meliloti* strains carrying MoClo-pABC vectors were grown in selective TY medium until an OD_600_ of 1. Cells were washed in 0.9% NaCl, resuspended, and adjusted to 1.39 × 10^5^ cells/μl (equivalent to 1 ng DNA/μl after cell lysis). Cells were lysed by incubation at 95 °C for 15 min. qPCR was carried out in a qTOWER^3^ G Thermal Cycler (Analytic Jena, Germany) using the TaykonNo Rox SYBRMasterMix dTTP Blue Kit and hard-shell PCR 96-well plates (BIO-RAD catalog-no: HSP9635). Reactions were performed according to the manufacturers’ instructions in a 20-μl reaction volume. Relative copy number was calculated as described elsewhere [47].

## Results

### Identification of heterologous ECF switches for dual use in *E. coli* and in *S. meliloti*

Twenty ECF switches composed of ECF/promoter pairs originating from various bacterial species (Table S7) were tested as putative context-independent regulators in the α-proteobacterium *S. meliloti*. These switches were previously shown to function in the γ-proteobacterium *E. coli* in a highly orthogonal manner [8]. ECFs are usually named in a systematic way (ECFXX_YYY), where XX refers to the phylogenetic group and YYY represents a unique ID [13]. The same nomenclature is used for autoregulatory *ecf* promoters (P_*ecfXX*_YYY_). Since the 20 ECFs analyzed in our study all are from different phylogenetic groups, an abbreviated identifier indicating only the phylogenetic group is used for simplicity. The full identifiers from Staron et al. [13] and the new identifiers assigned in a recent phylogenetic classification [12] are listed in Table S8.

**Fig. 1.**
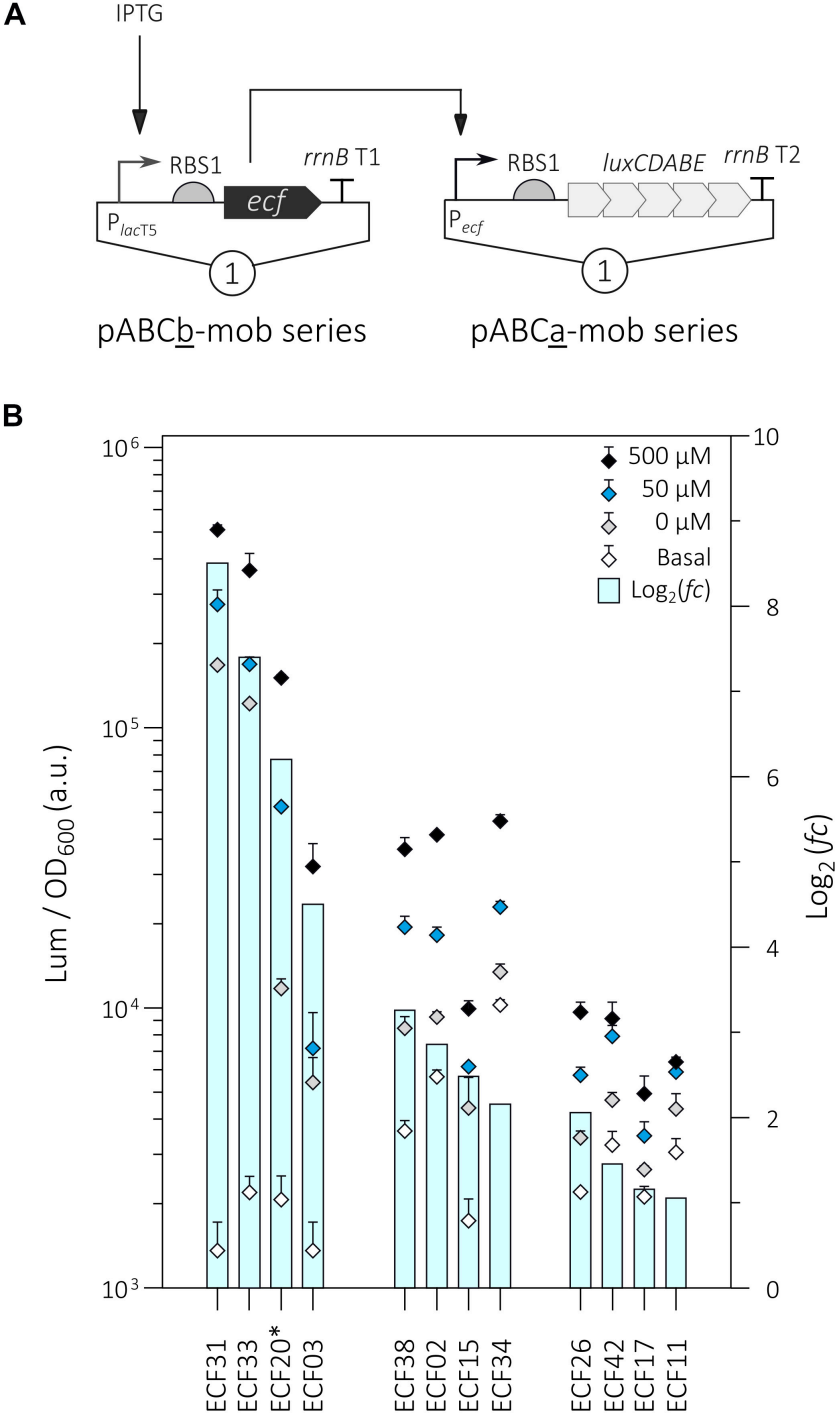
Evaluation of ECF-based genetic switches in *S. meliloti.* (A) Schematic representation of the experimental setup used to evaluate ECF switches in *S. meliloti*. Two single-copy pABC plasmid derivatives carry either transcriptional units for IPTG-dependent *ecf* expression (pABCb-mob) or the cognate *ecf* promoter (pABCa-mob) that is driving expression of a *luxCDABE* reporter. The P_*lac*T5_ promoter has been used as input promoter due to its high overall activity in the fully induced state (Fig. S1). RBS1 belongs to a group of strong RBS and *rrnB* T1 and *rrnB* T2 are efficient terminators in *S. meliloti* (Fig. S1). (B) Performance of heterologous ECF switches in the ECF/anti-σ-free *S. meliloti* strain*.* Different IPTG concentrations (0, 50, and 500 μM) were used to induce *ecf* expression. Promoter activities were normalized to yield luminescence units per unit of OD_600_ (left y-axis). To determine basal *ecf* promoter activities (basal), luminescence activity was assayed in strains carrying an empty pABCb-mob plasmid without any heterologous *ecf* gene. Each dot represents the mean response of 3 biological replicates 6 h after addition of IPTG. Arrow bars show standard deviation. Rectangular bars indicate the average log_2_(*fold change(fc)*) of promoter activity (right y-axis) in the presence of 500 μM IPTG compared to the basal promoter activity in the absence of any heterolougs *ecf*. The underlying data are given in Table S9. An asterisk (*) is indicative for an ECF with a 6xHis tag fusion at its N-terminus (Table S3).

The ECF switches were implemented in the *S. meliloti* Rm1021 wild type and the Rm1021-derived strain RFF625c, which is deleted for all native ECF- and anti-σ factor-encoding genes [48] and is referred to as ECF/anti-σ-free strain in this study. The latter strain was reported not to show any severe defects in growth or stress tolerance under laboratory conditions [48]. The 2 strains were chosen as genetic background to compare the performance of the heterologous ECF switches in the presence and absence of endogenous ECFs, potentially cross-activating the heterologous *ecf* promoters. Both components of the ECF switches, the *E. coli* codon-optimized ECF-encoding gene under control of an IPTG-inducible promoter and the target promoter fused to the *Photorhabdus luminescence luxCDABE* operon, used as reporter, were established on a 2-plasmid system (Fig. 1A and Table S3). This system utilized 2 combinable and mobilizable single-copy plasmid vectors of the *repABC*-based pABC family [32].

In this experimental setup, an increase in luminescence activity after induction of *ecf* expression was detected for 11 and 12 ECF switches in the Rm1021 wild type (Fig. S2) and ECF/anti-σ-free strain (Fig. 1B), respectively. This discrepancy is associated with the high basal activity of heterologous P*_ecf26_* that was only observed in the wild-type strain but not in the ECF/anti-σ-free strain. This observation will be experimentally addressed in more detail in the next section. Dose (0, 50, and 500 μM IPTG)-response characteristics of the active ECF switches during batch culture growth of the ECF/anti-σ-free strain are shown in Fig. S3. We grouped the ECF switches according to their dynamic range by comparing the on-state upon induction of *ecf* gene expression with 500 μM IPTG to the basal P*_ecf_* activity in absence of any heterologous *ecf* gene. Based on the deduced log_2_ ratios, 3 groups of regulators were distinguished: high-activity switches (log_2_(*fold change*(*fc*)) >4), medium-activity switches (log_2_(*fc*) from 2 to 4), and low-activity switches (log_2_(*fc*) between 1 and 2). The assignment of ECF switches to activity groups was similar in both host strains tested, with the exception of ECF26/P*_ecf26_*, for which reporter induction was detected in the ECF/anti-σ-free strain but not in the wild type (Fig. 1B and Fig. S2).

In our study, we intended to compare the behavior of synthetic genetic circuits composed of equivalent ECF/promoter pairs in single copy in *E. coli* and *S. meliloti* (see below). However, for the initial characterization of heterologous ECF switches in *E. coli*, multicopy plasmids were used to establish the *ecf*/P*_ecf_* unit [8]. A smaller subset has been chromosomally integrated for analysis in single copy by Pinto et al. [7]. Where necessary, we reanalyzed the performance of *ecf*/P*_ecf_* pairs integrated into the *E. coli* chromosome. This analysis was carried out for all *ecf*/P*_ecf_* pairs that showed activity in *S. meliloti* in our study, except for ECF38/P*_ecf38_*, ECF15/P*_ecf15_*, ECF34/P*_ecf18_*, ECF26/P*_ecf26_*, and ECF11/P*_ecf11_* which have been confirmed to be functional in single copy before [7]. The ECF02-dependent switch was omitted since this ECF (RpoE, σ^24^) is derived from *E. coli*, where it is involved in the cell envelope stress response [10]. We assembled the genetic parts of the remaining 6 ECF switches (*ecf* gene under control of the arabinose-inducible **P_BAD_ promoter and P*_ecf_*-*luxCDABE*) using CRIMoClo vectors and chromosomally integrated the resulting plasmids by site specific recombination into phage attachment sites in *E. coli* [18]. **P_BAD_ was used to drive *ecf* gene expression to allow comparison to the previously published design [7]. Functional characterization of the response dynamics of the chromosomally encoded ECF switches confirmed their activity in this genetic design in *E. coli* (Fig. S4), extending our previous findings [7]. Thus, the present analysis identified 11 ECF switches that are functional in both *S. meliloti* wild type and *E. coli*, demonstrating cross-species applicability of these regulators.

### Characterization of a set of heterologous ECF switches for crosstalk in *S. meliloti*

Interference with host components may limit the applicability of genetic modules for the construction of synthetic genetic circuits. We therefore tested the building blocks of the heterologous ECF switches for crosstalk with the host’s endogenous ECFs and the host genome, as well as for crosstalk with each other in the host context.

#### Endogenous ECFs can interfere with the performance of heterologous ECF switches from the same phylogenetic group

**Fig. 2.**
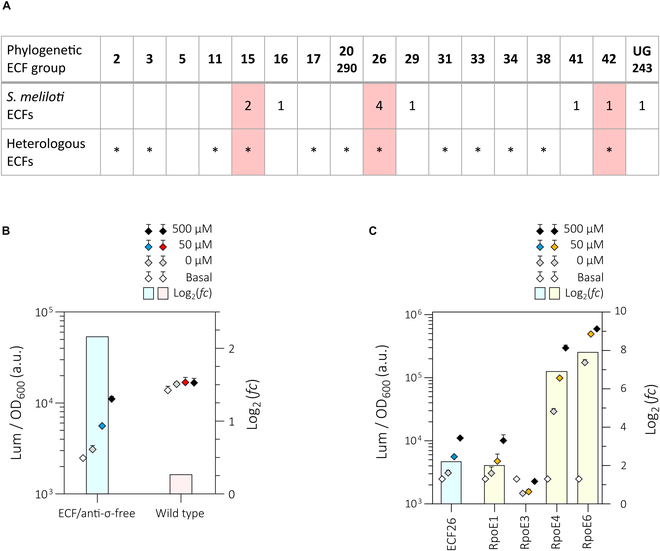
Endogenous ECFs can interfere with the performance of heterologous ECF switches. (A) The ECF profile of *S. meliloti* is compared to the heterologous ECF switches that were shown to be active in the Rm1021 wild type and/or the ECF/anti-σ-free strain. Phylogenetic groups are given on top. If 2 groups are listed, the first one refers to the initial classification [13] and the second one to the latest classification [12]. Numbers indicate the presence of one or more *ecf* genes from a certain phylogenetic group in the genome of *S. meliloti.* * is indicative of an active heterologous ECF switch. Red shaded boxes label shared phylogenetic ECF groups. (B) Performance of the ECF26-based switch in Rm1021 and the ECF/anti-σ-free strain and (C) P*_ecf26_* activation by endogenous ECFs from the same phylogenetic group (RpoE1, RpoE3, RpoE4, and RpoE6) assayed in the ECF/anti-σ-free strain. The experimental setup in (B) and (C) corresponds to the setup described in Fig. 1A. IPTG at different concentrations (0, 50, and 500 μM) was used to induce *ecf* expression. Promoter activities were normalized to yield luminescence units per unit of OD_600_ (left y-axis). Each dot represents the mean response of 3 biological replicates 6 h after addition of IPTG. Arrow bars show standard deviation. Rectangular bars represent the average log_2_(*fc*) of promoter activity in the presence of 500 μM IPTG compared to the basal promoter activity in the absence of any heterologous *ecf* (right y-axis). The underlying data are given in Table S9.

Inadvertent activation of heterologous *ecf* promoters by endogenous ECFs is likely if they belong to the same phylogenetic group as the cognate ECFs of the heterologous promoters. *S. meliloti* Rm1021 possesses 11 endogenous ECFs (RpoE1 to RpoE10 and FecI) [48,49]assigned to 7 different phylogenetic groups according to the most recent classification by Casas-Pastor et al. [12] (Fig. 2A and Table S8). ECFs of the phylogenetic groups 26 (4 ECFs), 15 (2 ECFs), and 42 (1 ECF) are shared by these endogenous ECFs and by the set of heterologous ECFs found to be active both in *E. coli* and the ECF/anti-σ-free *S. meliloti* strain (Fig. 2A). However, only the ECF26-based switch performed differently in the background of the *S. meliloti* wild type and the ECF/anti-σ-free strain under the conditions used here. A 6-fold higher basal promoter activity was detected in the wild type compared to the ECF/anti-σ-free strain (Fig. 2B). This suggests that cross-activation of the heterologous P*_ecf26_* promoter by endogenous group 26 ECFs increases basal promoter activity. This likely explains the lack of a further increase in reporter activity upon overexpression of the heterologous *ecf26* gene in the wild-type strain.

To determine whether the 4 endogenous group 26 ECFs (RpoE1, RpoE3, RpoE4, and RpoE6) are able to mediate activation of the heterologous P*_ecf26_* promoter, we assayed activity of the P*_ecf26_* reporter gene fusion upon induced expression of the respective pABC-borne endogenous *ecf* genes in the ECF/anti-σ-free strain. Induced *rpoE3* expression failed to activate P*_ecf26_*, while expression of *rpoE1* and the heterologous *ecf26* gene led to comparable activation patterns. The use of *rpoE4* and *rpoE6* resulted in even higher reporter activity compared to the heterologous *ecf26* gene (Fig. 2C). The average log_2_(*fc*) of promoter activity determined in the fully induced state was ~2 for ECF26, while RpoE4 and RpoE6 yielded a log2(*fc*) of ~7 and ~8 (Fig. 2C). Given the better performance of RpoE4, and its small regulon comprising less than 10 genes (including the *rpoE4* and *rpoE1* operon, which is mostly deleted in the ECF/anti-σ-free strain [48], RpoE4 can be used instead of the heterologous ECF26 in this *S. meliloti* strain. The response dynamic of the semiheterologous RpoE4/P*_ecf26_* switch is shown in Fig. S5. However, the same switch was not functional in *E. coli* (Fig. S5).

#### The host transcriptome is only minimally affected by heterologous ECFs

In addition to analyzing crosstalk of endogenous ECFs with heterologous ECF switches, we examined whether expression of heterologous ECFs alters the host’s transcriptome and whether alterations can be computationally predicted based on our knowledge of ECF/promoter specificities. For this analysis, we selected 2 ECFs from our set of ECF switches active in *S. meliloti* and not belonging to ECF groups represented in that host: ECF02 and ECF11 originating from the γ-proteobacterial species *E. coli* and *Vibrio parahaemolyticus*, respectively, representing a medium and weakly active ECF switch in *S. meliloti*. While ECF11 is more closely related to the endogenous ECFs RpoE1, RpoE3, RpoE4, RpoE6 of the ECF26 group, RpoE2 and RpoE5 of the ECF15 group, and RpoE7 of the ECF16 group, ECF02 is generally more distantly related to the *S. meliloti* ECFs [12]. However, ECF02 is known to have a large regulon in *E. coli* [50] making it an interesting candidate to analyze off-target transcription in a heterologous host. In order to identify off-target transcription from the host genome, *ecf11* and *ecf02*, under control of an IPTG-inducible promoter on multicopy plasmid pSRK Gm [m], and as control empty pSRK Gm [m] were introduced to the ECF/anti-σ-free strain.

**Fig. 3.**
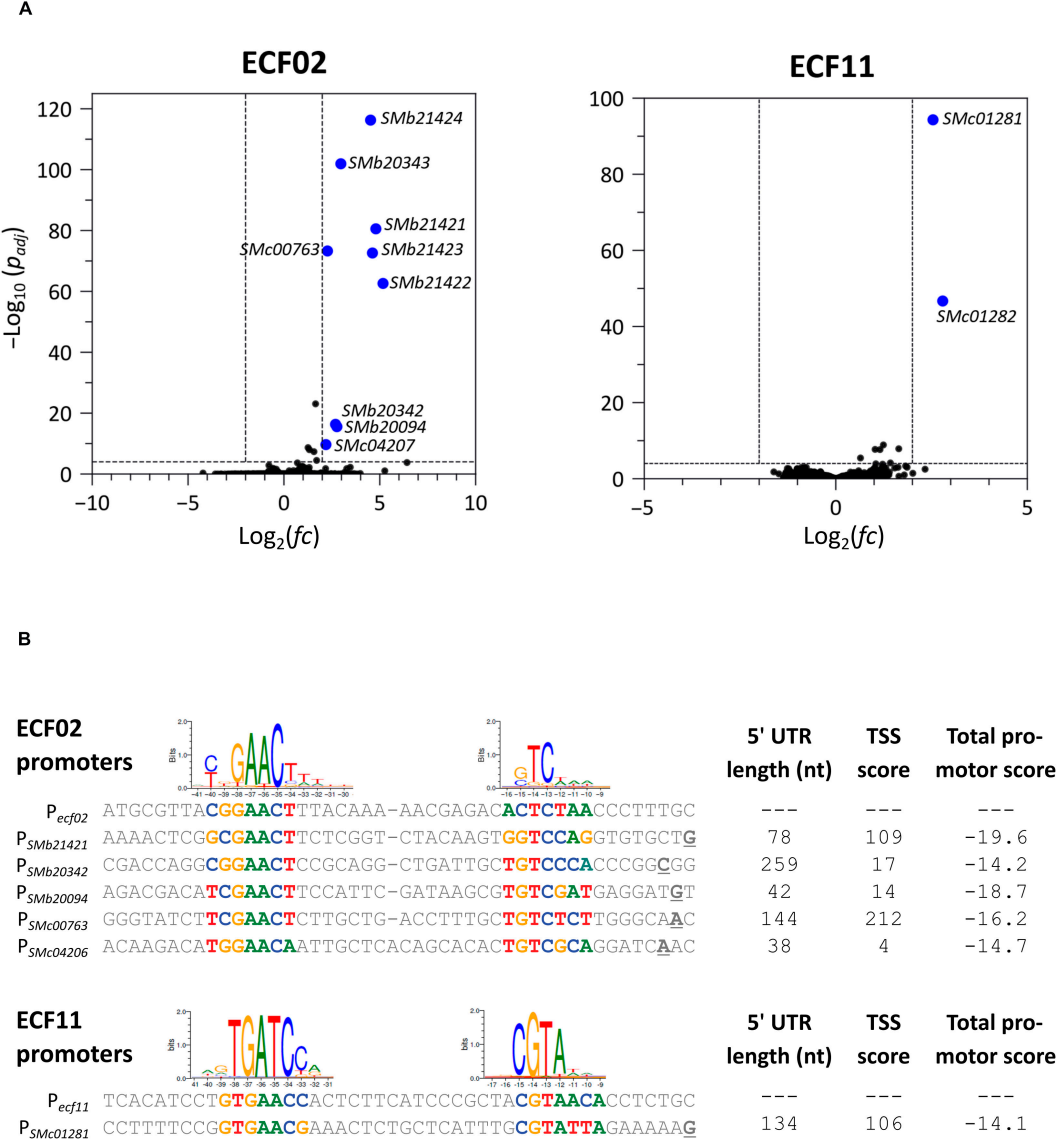
Accumulation of heterologous ECFs has minor effects on genome wide expression profiles. (A) Expression of *ecf02* and *ecf11* genes from the P_*lac*T5 _promoter on multicopy plasmid pSRKGm [m] was induced with 500 μM IPTG in the ECF/anti-σ-free *S. meliloti* strain. As control, a strain carrying empty pSRKGm was grown in presence of 500 μM IPTG. Assays were performed in triplicate. Data shown are derived from RNA-seq-based transcriptome analysis. Volcano plot representations of differential gene expression data (DEseq2-based, Table S6) are shown for *ecf02* and *ecf11* overexpression strains compared to the control strains. Significantly regulated genes (log2 fold change of >2 or <−2 and adjusted *P* values < 10^−4^) are highlighted in blue. (B) Cappable-seq was performed to identify ECF02- and ECF11-dependent TSS in the *ecf02* and *ecf11* overexpression strains. TSS with a score above 3.0 were identified upstream of all regulated genes/operons from A. P*_ecf02_*-and P*_ecf11_*-like promoter motifs could be assigned to the identified TSS (underlined, bold). Total promoter scores reflect similarities to ECF-specific promoter PSSMs [14]. For comparison, the heterologous P*_ecf02_* and P*_ecf11 _*promoters, and the ECF02 and ECF11 group-specific −35 and −10 consensus promoter motifs derived from the ECF hub [12] are shown. Length of the 5' untranslated region (5' UTR) is indicated.

We performed whole-transcriptome profiling by RNA-seq analysis of the *ecf02*- and *ecf11*-carrying strains and the control strain, grown under inducing conditions. Only a low number of significantly differentially expressed genes were detected in the *ecf*-overexpressing strains compared to the control (Fig. 3A). Overexpression of *ecf02* significantly increased the expression of 9 genes, presumably arranged in 5 operons (Table S10), at least by 4-fold. In the *ecf11* overexpression strain, 1 operon composed of 2 genes was significantly up-regulated (Table S10).

To support the identification of promoter sequences responsible for the observed ECF02- and ECF11-mediated transcriptional up-regulation, we mapped transcription start sites (TSS) on the genomic level by Cappable-seq [44] using the same RNA samples that were previously subjected to RNA-seq. Initially, we scanned sequences upstream of the regulated genes or operons for the presence of TSS. Notably, a TSS that was exclusively detected in the *ecf11* overexpression strain, was present upstream of the regulated *SMb21421-21424* operon. Similarly, strain-specific TSS were identified upstream of the *SMb21421-21424* and *SMb20342*-*20343* operons as well as upstream of the *SMb20094* and *SMc00763* genes in the *ecf02* overexpression strain but not upstream of *SMc04207* (Fig. 3B). Since *SMc04207* is likely arranged in an operon together with *Smc04206* and *SMc04208*, 2 genes which were also up-regulated in the RNA-seq experiment but just below the applied threshold of significance, we inspected sequences upstream of *SMc04206*, the first gene of that operon, and detected a strain-specific TSS as well. Noteworthy, P*_ecf02_*- and P*_ecf11_*-like promoters were identified closely upstream of these *ecf* overexpression strain-specific TSS (Fig. 3B), suggesting that the accumulation of heterologous ECFs is directly responsible for the observed differences in gene expression from the host genome.

Our previous analysis focused on ECF02- and ECF11-dependent expression of genes encoding annotated proteins or RNAs of our *S. meliloti* model. However, features missing in the genome annotation, transcriptional events from less conserved promoter sequences [51,52], and abortive transcription [53] may be responsible for further transcription mediated by the heterologous ECFs. To also detect such events, we identified ECF02- and ECF11-mediated transcription initiation events by comparative analysis of the *ecf*-overexpression strains with the control strain. We used a read length of 75 nucleotides resulting from our sequencing protocol and a TSS score above 3.0 (see Materials and Methods for details). Due to this read length, the detected TSS do not include the most common form of abortive transcription, as this leads to RNAs smaller than 23 nucleotides [53]. This comparative analysis identified 2,489 and 1,781 TSS specific to the *ecf02* and *ecf11* overexpression strains, respectively (Table S6). Likewise, 1,772 and 1,611 TSS specific to the control strain were detected, indicating similar variances across samples (Table S6).

To explore whether the *ecf* overexpression strain-specific TSS are associated with putative *ecf*-like promoters, upstream sequences of these TSS were analyzed for −35 and −10 motifs based on ECF-specific promoter PSSMs [14]. Table S6 lists these *ecf-*like promoters with *E*-values less than or equal to 0.05, along with total promoter scores based on similarity of −35 and −10 motifs with promoter PSSMs. In the *ecf11* overexpression strain, we identified 14 promoters with better total promoter scores than the one associated with the up-regulated operon in the RNA-seq data (Fig. 3B). In the *ecf02* overexpression strain, we used the promoter linked to *SMb21421-214224* for benchmarking because it displayed the worst score of the promoters associated to the ECF02-dependent genes derived from the RNA-seq data (Fig. 3B). One hundred thirty potential P*_ecf02_*-like promoters with better scores than the benchmarking promoter were found upstream of *ecf02* overexpression strain-specific TSS.

By computationally predicting the potential of off-target transcription mediated by heterologous ECFs, these kind of regulators could be more effectively used in a new bacterial host. We therefore tried to determine the crosstalk potential for ECF02 and ECF11 in silico and used ECF-specific promoter PSSMs to score sequences 500 bp upstream to 100 bp downstream of all annotated genes in the Rm1021 genome (see Materials and Methods for details). We predicted *ecf* promoters with the potential to promote off-target transcription upstream of 675 and 1,066 genes in the *ecf02* and *ecf11* overexpression strain, respectively. Notably, the in silico predicted promoters associated with the regulated genes in our RNA-seq experiment overlap with the ones we identified based on the TSS data. Figure S6 illustrates the distribution of total promoters from all in silico predicted promoters in the *ecf02* and *ecf11* overexpression strain. The promoter associated with the regulated *SMc01281-01282* operon in the *ecf11* overexpression strain has a better score than the mean score of all computationally predicted off-target promoters. The same is true for 3 out of 5 promoters associated with up-regulated genes in the *ecf02* overexpression strain.

#### Heterologous ECFs show weak albeit similar crosstalk patterns in different bacterial host species

**Fig. 4.**
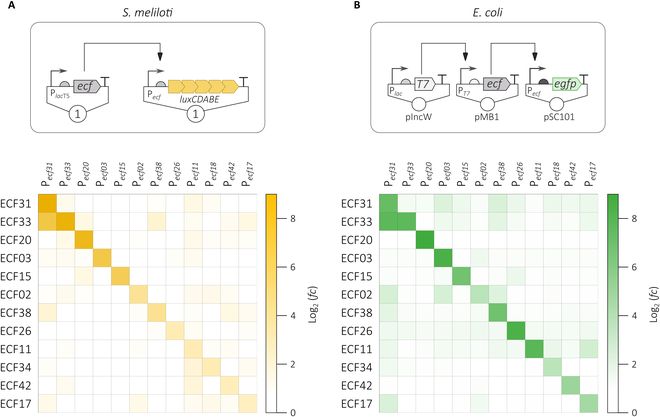
Cross-activation of heterologous *ecf* promoters by heterologous noncognate ECFs in *S. meliloti*. (A) From the set of 12 ECF switches found to be active in the ECF/anti-s-free *S. meliloti* strain, each of the 12 *ecf* promoters was tested for activation by each of the 12 ECFs using the depicted 2-plasmid-based experimental approach that was described in detail in Fig. 1A. Expression of the ecf gene was induced with 500 μM IPTG. P*_ecf_* activity was determined during exponential growth as relative luminescence units normalized by OD_600_. Each square represents the average log_2_(*fold change*) of promoter activity compared to a strain carrying P*_ecf_* in the absence of any heterologous ECF. Promoter activation was determined based on 3 biological replicates 6 h after addition of IPTG. The underlying data are given in Table S9. (B) Orthogonality of the same subset of ECF switches in *E. coli* using a multicopy plasmid-based assay system. Data were compiled from [8].

Construction of ECF-based genetic circuits requires combination of multiple ECF-based switches. This is only possible with high specificity of the ECFs for their cognate promoter and low cross-activation of noncognate promoters. Activation of *ecf* promoters across different ECF groups has been extensively mapped in *E. coli* and was exceptionally low [8]. Because we added *S. meliloti* as another chassis for ECF switches derived from this previous study, and also changed the experimental setup, we examined whether expression of any of the heterologous *ecf* genes found to be active in the ECF/anti-σ-free strain results in unexpected activation of any of the heterologous nontarget promoters tested. We found that the ECF switches analyzed in *S. meliloti* and in *E. coli* showed similar orthogonality (Fig. 4).

Remarkably, the most prominent cross-activation of P*_ecf31_* by ECF33 occurred in both bacterial species. Dose-response curves of the hybrid ECF33/P*_ecf31_* switch showed good performance in *E. coli* and *S. meliloti* with respect to the dynamic range between inducing and noninducing conditions (Fig. S7).

### Comparison of analogous ECF-based genetic circuits in *S. meliloti* and *E. coli*

ECF switches have been combined in a recent study to create cascaded circuits for more complex applications, allowing a tunable delay between inducer addition and target gene activation [7]. With the set of ECF switches functional in *E. coli* and *S. meliloti* at hand, we compared ECF switch-based 2-step delay circuits in both hosts in single copy, further demonstrating the scalability of this approach across species. These delay circuits were composed of an input module including an *ecf* gene (named *ecfX*) under control of an inducible promoter (P*_ind_ ecfX*), a delay module consisting of a further *ecf* gene (named *ecfY*) under control of a target promoter of ECFX (P*_ecfx_ ecfY*), and an output module comprising *luxCDABE* under control of a target promoter of ECFY (P*_ecfy_ luxCDABE*). The delay module acts as connector of the input and output module since accumulation of the encoded ECF activates expression of the *luxCDABE*-mediated output. This configuration was chosen for reasons of comparability, as it has been used previously in *E. coli* and *B. subtilis* [7]. In *E. coli*, as in the previous work, the synthetic genetic circuit was implemented in single copy by integration into the chromosome. In *S. meliloti*, however, it was established on one or multiple pABC-based single-copy plasmids [32].

#### MoClo-compatible pABC plasmids facilitate modular construction of synthetic genetic circuits in *S. meliloti*

**Fig. 5.**
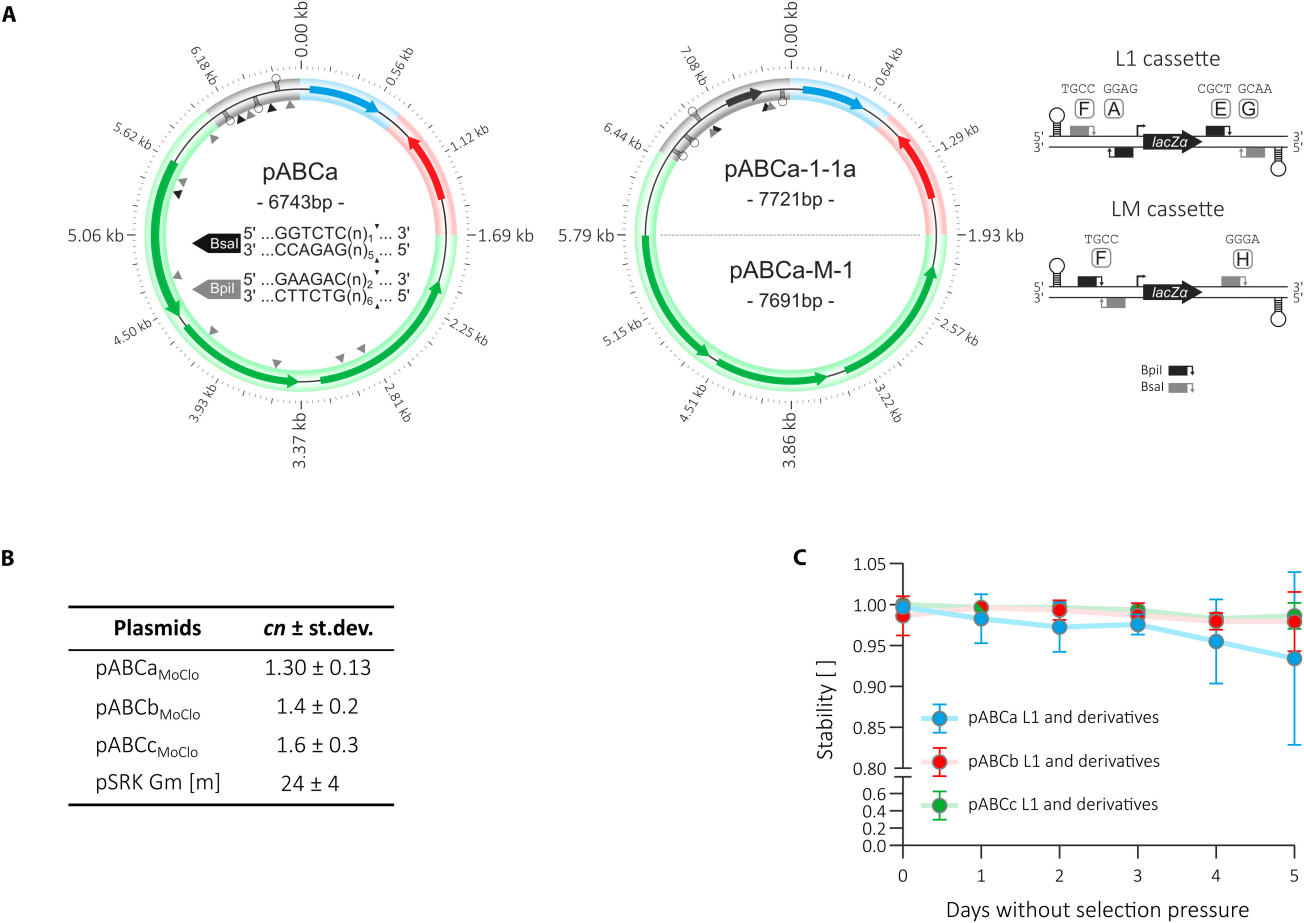
Functional analysis of MoClo pABCs in *S. meliloti*. (A) Exemplary shown are schematic representations (drawn to scale) of pABCa [32] and MoClo-compatible pABCa-1-1a and pABCa-M-1 plasmids. Arrows represent open reading frames or plasmid origins for the use in *E. coli*. Colors refer to specific module parts: oriVSm (green), oriVEc (blue), AR (red), synterMCS (gray). BpiI and BsaI recognition sites are highlighted by gray and black triangles, respectively. All module parts of pABCa-1-1a and -M-1 were cleared for the 12 BpiI/BsaI sites present in pABCa. Level 1 and level M cloning cassettes were derived from vectors pICH47732 [25] and pAGM8031 [54]. The orientation of BpiI and BsaI recognition sites and the associated fusion sites, both represented by capital letters, are shown in more detail. (B) qPCR-determined copy number of MoClo pABCs in Rm1021. Cells were harvested at the exponential growth phase. Measurements are based on 4 biological replicates (Table S9). As a control, copy number of pSRKGm [34] was determined in Rm1021. (C) Propagation stability of MoClo-compatible pABC plasmids. Propagation stability was assayed in *S. meliloti* Rm1021 strains DMSm107, DMSm108, and DMSm109, harboring 3 pABC-Level1 plasmids or derivatives, respectively. Strains were maintained at exponential growth (OD_600_ = 0.05 to 1) over a period of 5 d. Samples were collected every day. Single colonies (*n* = 96) were examined for antibiotic resistance against gentamicin, hygromycin, and kanamycin (indicative of the presence of pABCa-1-1a, pABCb-mod-1-1a, and pABCc-1-1a or derivatives). Error bars represent standard deviation of 3 biological replicates.

The part library for construction of ECF switches and ECF switch-based regulatory circuits has been established using the Golden Gate DNA assembly standard MoClo, which uses the type IIs endonucleases BpiI and BsaI [25]. This assembly standard was not applicable to the recently published pABC vector family [32] since the pABCa-c vectors contain 12, 5, and 15 BpiI or BsaI recognition sites, respectively (Fig. 5A and Fig. S8). Therefore, for construction of ECF switches, input and output modules had to be assembled into standard vectors of the MoClo system before they could be recloned into pABCs using conventional methods.

To facilitate modular gene circuit construction on pABC vectors we constructed a subset of MoClo-compatible pABC vectors for use in *S. meliloti*. To this end all parts of the modular pABCa-c vectors (carried by library plasmids, Table S3) were cleared for all BpiI and BsaI recognition sites. These included *repABC* cassettes (oriVSm) mediating replication and segregation in *S. meliloti*, plasmid-derived origins of replication (oriVEc) for propagation in *E. coli*, antibiotic resistance genes (AR), multiple cloning sites flanked by transcription terminators (synTer-MCS), and a RK2/RP4 mobilization site (*mob*) for plasmid transfer by conjugation (Table S3). The synTer-MCS was further equipped with a *lacZα* fragment for blue-white screening flanked by BpiI/BsaI restriction recognition sites as determined by the MoClo standard.

Based on these BsaI/BpiI-cleared library plasmids, we generated a set of 11 MoClo-compatible pABCa-c derivatives that allow Level 1 and Level M assemblies (Table).

**Table.** Characteristics of MoClo-compatible pABC plasmids generated in this study and comparison to pABCs from the original set [32]. Analogous pABC plasmids from the original and the MoClo-compatible set always share the same oriVSm and oriVEc module. The AR resistance of some MoClo pABCs differs from the original counterpart in that Sp and Km resistances were avoided. To highlight changes in antibiotic resistance cassettes, a bold typeface is used. This allows higher-order DNA assemblies using LevelM or Level2 plasmids from Weber et al. [25] and Werner et al. [54] that carry a spectinomycin and a kanamycin resistance gene, respectively. The introduced L1 and LM cloning cassettes shown in bold typeface correspond to plasmids pICH47732 [25] and pAGM8031 [54]. They are part of the synter MCS module and show high sequence similarity due to the presence of a *lacZα* fragment. This gene fragment is lost after successful assembly reactions.

	pABC plasmids	oriVSm (*oriV* origin)	oriVEc (*oriV* origin)	synter MCS cassette		AR (resistance)	oriT (*mob* site)	Plasmid size (bp)
Original set of pABCs	pABCa	pMlb	p15A	*synTer1*		Gm	-	6,743
	pABCb	p42b	pMB1	*synTer2*		Sp	-	6,545
	pABCc	p42d	pSC101*	*synTer3*		Km	-	11,514
	pABCa-mob	pMlb	p15A	*synTer1*		Gm	*mob*	7,207
	pABCb-mob	p42b	pMB1	*synTer2*		Sp	*mob*	7,009
	pABCc-mob	p42d	pSC101*	*synTer3*		Km	*mob*	11,978
MoClo-compatible pABCs	pABCa-1-1a	pMlb	p15A	*synTer1*	**L1**	Gm	-	7,721
	pABCa-M-1	pMlb	p15A	*synTer1*	**LM**	Gm	-	7,691
	pABCb-mod-1-1a	p42b	pMB1	*synTer2*	**L1**	**Hyg**	-	7,757
	pABCb-M-1	p42b	pMB1	*synTer2*	**LM**	Sp	-	7,493
	pABCc-1-1a	p42d	pSC101*	*synTer3*	**L1**	Km	-	12,495
	pABCc-M-1	p42d	pSC101*	*synTer3*	**LM**	Km	-	12,465
	pABCc-mod-1-1a	p42d	pSC101*	*synTer3*	**L1**	**Tet**	-	12,892
	pABCa-1-1a-mob	pMlb	p15A	*synTer1*	**L1**	Gm	*mob*	8,251
	pABCa-M-1-mob	pMlb	p15A	*synTer1*	**LM**	Gm	*mob*	8,221
	pABCb-mod-1-1a-mob	p42b	pMB1	*synTer2*	**L1**	**Hyg**	*mob*	8,287
	pABCb-M-1-mob	p42b	pMB1	*synTer2*	**LM**	Sp	*mob*	8,023

To compare the functionality of these MoClo-adapted parts with the parental parts, we determined the copy numbers of pABCa-1-1a, pABCb-1-1a, and pABCc-1-1a vectors or derivatives under selective conditions by qPCR (Fig. 5B). Further, we tested reliable vertical plasmid propagation without antibiotic selection over a 5-d period with daily reinoculation, showing similar properties between parental and MoClo adapted vectors (Fig. 5C).

For analysis of plasmid propagation stability each of these 3 MoClo-compatible pABC vectors was combined with 2 derivatives lacking the *lacZα* cassette to avoid possible homologous recombination events between the 3 vectors via this region in this assay (Fig. 5B). At the end of this experiment, loss of any of these plasmids—indicated by antibiotic sensitivity—was detected for only 7% (pABCa-1-1a or derivatives), 2% (pABCb-1-1a or derivatives), and 1% (pABCc-1-1a or derivatives) of the colonies, and none of these colonies showed loss of more than one of the 3 pABCs. The MoClo-compatible pABCs expand the original pABC family. For the design of synthetic genetic circuits all a-, b-, and c-type pABCs are freely combinable in *S. meliloti* due to compatible *repABC*-type replication and segregation regions.

#### Single-copy number delay circuits implemented in *S. meliloti*

**Fig. 6.**
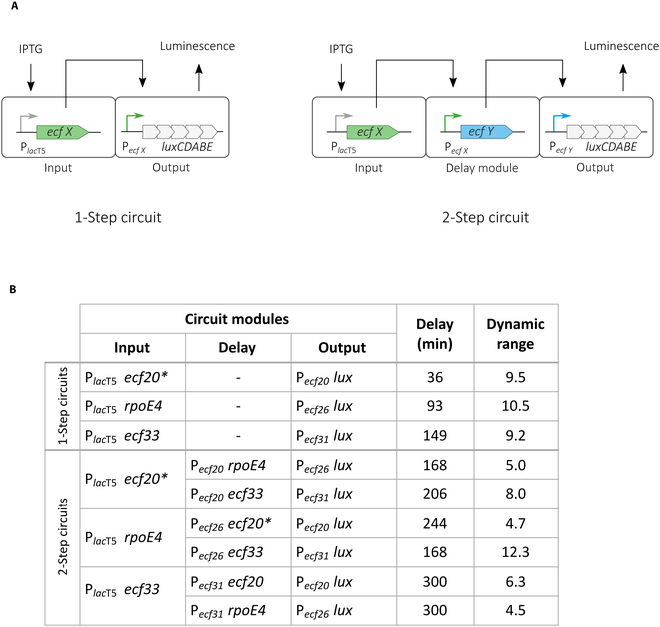
ECF-based delay circuits in the ECF/anti-σ-free *S. meliloti* strain. (A) Schematic representation of 1-step and 2-step timer circuits. The input module is composed of an inducible P_*lac*T5_ promoter for IPTG dependent *ecfx* expression. The output module contains a suitable *ecf* promoter for *luxCDABE* expression. 2-step circuits include a P*_ecfX_* *ecfY* delay module, which connects input and output aiming at delayed *luxCDABE* expression. Input, delay, and output modules are carried by pABCb-mob, pABCc-mob, and pABCa-mob plasmids, respectively. Detailed assemblies of transcription units are described in Table S3. (B) Main characteristics of 2-step timer circuits implemented in the ECF/anti-s-free *S. meliloti* strain. For comparison, time delay and the dynamic range of the parental 1-step timers are shown. The respective *S. meliloti* strains carry an empty pABCc-mob plasmid without a delay module. The time delay is shown for the highest IPTG concentration (500 μM). It is determined as time period after IPTG addition, until the average luminescence exceeded the basal luminescence level (measured in the absence of IPTG) by at least 2-fold. The dynamic range was determined 18 h after the addition of IPTG as average fold increase of the basal luminescence. All measurements were carried out at least in biological triplicates. An asterisk (*) is indicative for an ecf gene that has been translationally fused with a 6xHis tag encoding sequence (Table S3).

Two-step timers (Fig. 6A) were constructed using the ECF switches ECF20*/P*_ecf20_*, RpoE4/P*_ecf26_* and ECF33/P*_ecf31_* since these displayed the highest fold induction of reporter gene expression when compared to noninducing conditions (~10 fold) (Figs. S3, S5, and S7). For the input and output modules, the pABCb-mob and pABCa-mob plasmid series was used, respectively (Fig. 1A). The delay module was established on pABCc-mob. We implemented all 6 possible combinations of 2-step timers in *S. meliloti* and compared the dynamic behavior of the cascades to strains with the respective 1-step *ecf* gene circuits, composed of P*_ind_ ecfx* and P*ecf_x_ luxCDABE* but lacking the delay module. For simplicity, these switches are referred to as 1-step timers.

The dynamic range and the time delay of reporter gene expression were specified for each 1-step and 2-step ECF circuit (Fig. 6B). Detailed dose-response curves are shown in the supplement (Fig. S9). For all implemented 2-step timers, the delay between IPTG addition and the time point when luminescence exceeded the basal level by at least 2-fold was longer than for the parental 1-step timers. For instance, the time delay of *ecf20-* and *rpoE4*-based 1-step timers at 500 μM IPTG was 36 and 93 min, respectively, while the time delay of the 2-step timers was 168 min (P_*lac*__T5_ ecf20* as input) and 244 min (P_*lac*_*_T5_ rpoE4* as input). The dynamic range, determined as the fold induction of basal luminescence 18 h after addition of IPTG, slightly decreased as compared to the individual 1-step timers. Generally, our results are in accordance with the experimental and computational modeling data obtained for similar ECF-based 2-step timers in *E. coli* and *B. subtilis* [7].

#### Analogous single-copy ECF circuits are functional in *S. meliloti* and *E. coli*

For comparison of analogous 2-step timers composed of identical ECF/P*_ecf_* pairs in *S. meliloti* and in *E. coli*, we selected *ECF20/P*_ecf20_*, and ECF33/P*_ecf31_* since both switches performed well in terms of combinability and dynamic range in both hosts (Figs. S3, S4, and S7). For *E. coli*, these switches were combined into the 2-step timers in both possible orders. Since we adopted the established circuit design from Pinto et al. [7], the arabinose inducible P_BAD_ promoter was used to drive expression of the input ECF-encoding gene. The genetic input, delay, and output modules insulated by arrays of transcriptional terminators and carried by the CRIMoClo vector pSV006 were chromosomally integrated by site-specific recombination at the P21 phage attachment site [18] (Fig. S10)**.**

**Fig. 7.**
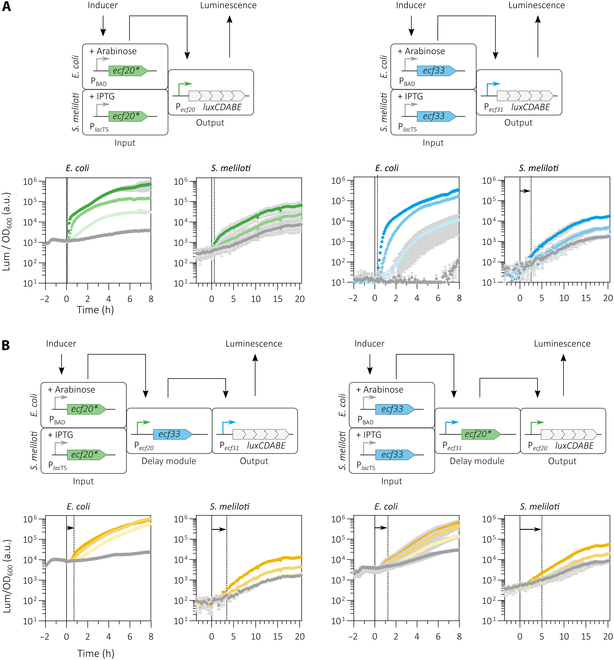
Performance of analogous 1-step (A) and 2-step (B) delay circuits in *S. meliloti* and *E. coli*. The genetic constructs of all circuits were either integrated into the chromosome of *E. coli* SV01 or carried by single-copy number pABCa-c mob derivatives in the ECF/anti-σ-free *S. meliloti* strain. Panels show the response of luciferase activity (relative luminescence normalized by OD_600_). Expression of *ecf* genes were induced with 0, 10^−5^, 5 × 10^−5^, and 2 × 10^−1^% of arabinose (*E. coli*) or with 0, 5, 50, 500 μM IPTG (*S. meliloti*) at *t* = 0 h (black solid line). The basal luciferase activity (absence of any inducer) is shown as gray solid line. Colored lines in shades of green, blue (1-step timers), and yellow (2-step timers) show the specific response after induction of *ecf* expression with increasing inducer concentrations. Error bars represent standard deviations of at least 3 biological replicates. Underlaying data can be extracted from Table S10. Dashed lines indicate the time delay after addition of maximum inducer concentrations until the average luminescence exceeded the basal luminescence level by at least 2-fold. The maximum dynamic output range (*dr*) was determined 8 h (*E. coli*) and 18 h (*S. meliloti*) after inducer addition. An asterisk (*) is indicative for an *ecf* gene that has been translationally fused with a 6xHis tag encoding sequence (Table S3). Detailed information on assembled transcription units can be extracted from Table S3.

Both the *ECF20- and ECF33-based 1-step timers (Fig. 7A) and the 2 derived 2-step timers (Fig. 7B) showed characteristic inducer concentration- and time-dependent output signals in *E. coli* and *S. meliloti*, despite different implementation strategies—chromosomal integration in *E. coli* and introduction on single-copy plasmids in *S. meliloti*. However, the dynamic range of both 1-step and 2-step timers was larger in *E. coli* than in *S. meliloti*. This difference can be explained by the exceptional performance of the P_BAD_ promoter in *E. coli*, which showed a ~50,000-fold induction of luciferase activity upon addition of the inducer [7].

#### Multigene circuits in single copy on a single plasmid or distributed on multiple plasmids show similar properties in *S. meliloti*

**Fig. 8.**
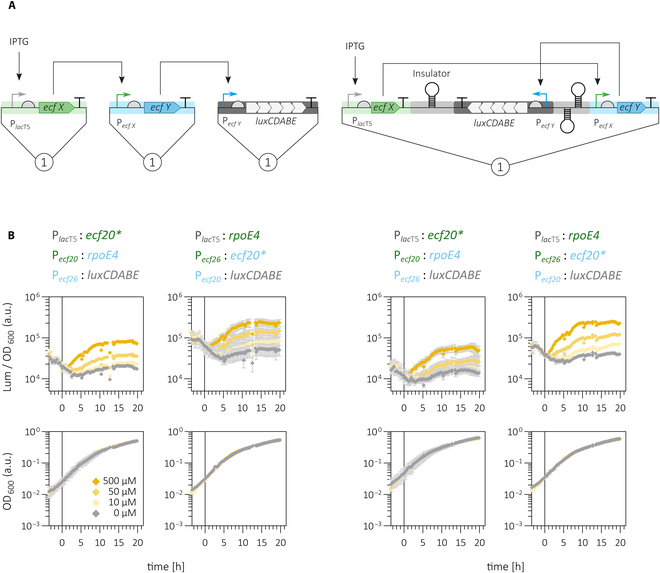
Performance of multitranscriptional unit gene circuits in *S. meliloti* using different experimental setups. The performance of ECF-based 2-step timer circuits encoded by genetic constructs on 3 or a single pABC plasmid was assayed in the ECF/anti-σ-free *S. meliloti* strain. (A) Schematic representation of the experimental layout. Input, delay, and output modules of the 2-step timers were either encoded by 3 single-copy number vectors (pABCa-mob, pABCb-mob, and pABCc-mob backbones) or by a single pABCa derivative. When using the 1-plasmid approach, additional terminators were included for insulation of individual transcriptional units as detailed in Figs. S10 and S11 and in Table S3. (B) Time course of luciferase activity following induction of *ecfX* expression with 0, 10, 50, and 500 μM of IPTG at *t* = 0 h. Luciferase activity is shown as relative luminescence normalized by OD_600_ of the *S. meliloti* culture. Gray dashed lines represent noninducing conditions. Dashed lines in shades of yellow indicate increasing inducer concentrations. Error bars represent standard deviation of at least 3 biological replicates. An asterisk (*) is indicative for an ecf gene that has been translationally fused with a 6xHis tag encoding sequence (Table S3).

In our initial approach to implement single-copy-number 2-step timers in *S. meliloti*, we provided the input, the delay, and the output module on different pABC-mob plasmids that insulated the individual TUs on separate replicons (Fig. 8A). For comparing this 3-replicon design to a 1-replicon design, we assembled two 2-step timers composed of *rpoE4*/P*_ecf26_* and *ecf20/P*_ecf20_* switches in the MoClo-compatible pABCa-M1-mob vector (Fig. 8A). To avoid transcriptional interference, input, delay, and output modules were insulated by arrays of terminators (Fig. S11). The layout of the 2-step delay circuits on 3 or on a single pABC plasmid did neither affect growth of *S. meliloti* nor did it substantially change the time delay and the response dynamics (Fig. 8B). Growth factors were determined for strains carrying 2-step delay circuits employing both experimental setups as well as for strains carrying the parental 1-step timers revealing no notable differences (Table S12).

## Discussion

### Proteobacteria have a similar phylogenetic acceptance range for heterologous ECFs

We showed that heterologous ECF switches that function in the γ-proteobacterium *E. coli* [8] can be transferred to the α-proteobacterium *S. meliloti* with a success rate of more than 50%, and that these switches also maintained their pattern of orthogonality in both hosts. These include ECFs from α-, and γ-proteobacteria, e.g. *Caulobacter crescentus* (ECF41) or *Pseudomonas fluorescencs* (ECF20), as well as from different classes of bacteria, such as Firmicutes (ECF31 from *B. subtilis*), Bacteroidetes (ECF03 from *Bacteroides thetaiotaomicron*), and Actinobacteria (ECF38 from *Streptomyces coelicolor*). ECF switches that were not active in *S. meliloti* in our study comprise ECFs from α-, β-, and γ-proteobacteria and from the Actinobacterium *S. coelicolor*. Moreover, ECFs derived from the same bacterial species showed different results in *S. meliloti*. For example, 2 of the 4 *S. coelicolor* ECFs tested, ECF34 and ECF38, were found to be active, whereas the others (ECF14 and ECF27) failed to measurably activate the provided promoter. A similar observation was made for ECF42 and ECF22 from *Xanthomonas campestris*, which were found to be active and inactive, respectively, in *S. meliloti*. Hence, phylogenetic relatedness between the ECF donor strain and *S. meliloti* may play a role but is probably not the major factor for ECF activity. Other factors that contribute to the functionality of ECF switches are probably *ecf* transcription and mRNA translation rates, and ECF protein stability in the heterologous host, as well as host-specific factors that influence the transcription initiation rate of the target promoter.

In contrast to the large overlap in activity of phylogenetically distinct heterologous ECFs in our model γ- and α-proteobacterial hosts, studies in *B. subtilis* showed a rather narrow acceptance range for heterologous ECFs. From 46 ECF switches tested, only 4 were active. Three of these active switches were derived from other members of the genus *Bacillus* and 1 originated from *Streptomyces venezuelae* [17]. This implies that even though core RNAP subunits and interactions between RNAP and σ factor exhibit a high degree of conservation across bacteria [15,16], lineage-specific variations of the RNAP core or the sigma factors could influence the acceptance range for heterologous ECFs. In a recent study, Forrest et al. [55] identified at least striking differences between the housekeeping sigma factor in *B. subtilis* (σ^A^) and in *E. coli* (σ^70^). According to these results, σ^A^ has a stronger preference for AT-rich discriminator sequences at promoters and a narrower acceptance range for variations in its length [55]. The observation that *E. coli* and *S. meliloti* have a broad phylogenetic acceptance range of ECFs suggests that their use as context independent regulators can presumably be expanded into other proteobacterial species. The collection of ECF/promoter pairs carried in level 0 MoClo standard plasmids developed here and in our previous studies [7] can serve as suitable starting point.

### Performance of ECF regulatory switches in *S. meliloti*

The 12 active ECF switches in *S. meliloti* have been characterized in single copy using 2 pABC plasmids that carry either the IPTG-inducible *ecf* gene or the *ecf* target promoter driving *luxCDABE* expression. When comparing the fully induced to noninduced states, the dynamic range of the best performing switches was about 10-fold and of the worst performing switches about 4-fold. In *B. subtilis*, heterologous ECF switches are similarly active when the transcription units are integrated in single copy at different genomic loci [37]. However, comparing P*_ecf_*-reporter activity between an *S. meliloti* strain carrying the complete ECF switch and a strain carrying the reporter construct but lacking the *ecf* gene, the maximum induction was up to 1,000-fold. The discrepancy is due to the leakiness of the P_*lac*T5_ promoter, which leads to substantial *ecf* expression in the absence of IPTG. However, well-characterized inducible promoters with low background activity and high dynamic range are still lacking for *S. meliloti*. This is different in *E. coli*. Here, the low basal activity of the arabinose-inducible P_BAD_ promoter driving *ecf* expression allowed for a 20,000-fold induction of target gene expression, and remarkably, the output signal of strains with the P*_ecf_*-reporter construct combined with or without P_BAD_-*ecf* showed no difference [7]. With the implementation of a set of ECF switches in *S. meliloti*, we expand the available genetic tools for this plant symbiotic bacterial species. However, to exploit the full potential of these regulators, input promoters need to be improved.

### Preselection of heterologous ECF switches with minimal crosstalk potential in a new host

To ensure optimal function of heterologous ECFs, crosstalk between these regulators or crosstalk in the host context should be minimized. ECF switches analyzed in *E. coli* [8] and in *S. meliloti* showed similar crosstalk-patterns for the activation of noncognate *ecf* promoters, highlighting the crucial role of sequence-specific contacts between the DNA and the ECF for promoter selectivity [14]. It is likely that these ECF/promoter pairs will maintain their orthogonal behaviors also in other bacterial hosts. Thus, experimental data obtained in one host seem to be transferable to other hosts, facilitating a preselection of suitable ECF/promoter pairs for new hosts. Moreover, computational predictions of cross-recognition of promoters by different ECF regulators as deduced from individual promoter preferences have also been shown to be highly reliable [14]. A recently published ECF hub database provides information on a huge number of ECFs and their phylogenetic relationships [12]. Both, this database, and computational predictions of promoter-specificity supports the preselection of multiple ECF/promoter pairs to be combined in one bacterial host and choosing suitable candidates for circuit design in new bacterial chassis. This preselection is recommended to also aim for regulators from ECF families with no member present in the new host to reduce the risk of interference with the endogenous ECFs.

Furthermore, prediction of off-targets of heterologous ECFs in the host genome would be desirable. Our computational analysis predicted many more *ecf*-like promoters in the *S. meliloti* genome than heterologous *ecf* expression-associated up-regulated genes identified by our whole-transcriptome analysis. Nonetheless, TSS mapping indicated transcriptional activity beyond these up-regulated genes, with approximately half of these TSS associated to computationally predicted *ecf*-like promoters. However, many of these promoters were predicted with rather low confidence. A precise prediction of off-targets for heterologous ECFs based on the parameters used does not seem possible. However, since a general correlation was observed between the number of predicted promoters, and the number of ECF-dependent up-regulated genes and identified TSSs, such prediction could nevertheless contribute as a rough proxy to preselecting heterologous ECF/promoter pairs with a low number of off-target sites in the host genome. For a detailed picture under the desired growth conditions, however, experimental validation is still required. The presence of additional promoter features, e.g. A/T-rich UP elements upstream of the −35 element [56] or pyrimidines and purines at the −1 and +1 position [57] might be necessary in some cases to initiate transcription at significant rates. More knowledge about such additional features required for transcription initiation from specific ECF promoters may inform future improved computational off-target predictions.

### Single-copy plasmid vectors for modular assembly of genetic circuits in *S. meliloti*

Previously, we have established the pABC family of shuttle plasmid vectors that replicate in single copy in members of the α-proteobacterial order *Hyphomicrobiales* (Rhizobiales) and in multicopy in *E. coli* [32,58]. Here, we demonstrated that these plasmids are an attractive platform for stable modular assembly of genetic circuits on multiple replicons, which eliminates the need for chromosomal integration.

Standardization of DNA assembly is an important feature that facilitates genetic engineering in synthetic biology [59]. The original standard for assembling pABC vectors from a library of parts is based on the ligase chain reaction and a set of standardized bridging oligonucleotides [31,32]. Application of the widely used Golden Gate modular cloning (MoClo) DNA assembly based on a set of Type IIS restriction enzymes [25] was previously hindered by the presence of recognition sites for these restriction enzymes in pABC parts. Particularly the *repABC* parts, which mediate replication and segregation in the *Hyphomicrobiales* hosts [32] contain a high number of these sites, making their removal challenging without impairing the important *repABC-*mediated functions. In this study, we succeeded in establishing a set of functional parts free of MoClo-specified Type IIS restriction enzyme recognition sites for all pABC modules for application in *S. meliloti*. This includes 3 *repABC* parts that allow 3 different pABC plasmids to be established together in *S. meliloti*. By providing a MoClo-based ECF toolbox and MoClo-compatible pABC vectors, we facilitate the use of an iterative “design-build-test” approach to analyze genetic circuits in single copy in *S. meliloti* and most likely in closely related members of the *Hyphomicrobiales*, in which the 3 parental *repABC parts* were functional, i.e., *Agrobacterium tumefaciens (Agrobacterium fabrum)*, *Mesorhizobium loti*, *Sinorhizobium fredii*, and *Rhizobium leguminosarum* [32].

## Supplementary Materials

Supplementary 1 Figs. S1 to S11 Tables S1 to S12 References [60–75]
