# Supplementary material for: A MoClo-Compatible Toolbox of ECF Sigma Factor-Based Regulatory Switches for Proteobacterial Chassis
Source: Biodes Res. 2024 Feb 21;6:0025. doi: 10.34133/bdr.0025 (PMC10880074; doi:10.34133/bdr.0025)
Supplement: Supplementary 1 — Figs. S1 to S11 Tables S1 to S12 References [60–75] [file bdr.0025.f1.zip › Supplementary_Materials.docx]

**Supplementary Materials**

**Table S1**. Bacterial strains. **(Separate file)**

**Table S2**. Primers and synthesized DNA. **(Separate file)**

**Table S3**. Plasmids. **(Separate file)**

**Table S4**. Gene expression comparisons. **(Separate file)**

**Table S5**. TSS mappings. **(Separate file)**

**Table S6**. ECF02/ ECF11 specific TSS and promoter predictions. **(Separate file)**

**Table S9.** Raw data points of main figures. **(Separate file)**

**Table S11.** Raw data points supplementary data. **(Separate file)**

**Table S7.** Origin and phylogenetic distribution (colour code) of heterologous ECF/promoter pairs (ECF switches) used in this study.

|  | **ECF σ** | **origin ECF σ** | **P*_ecf_*** | **origin P*_ecf_*** |
| --- | --- | --- | --- | --- |
|  | ECF20_0992 | *Pseudomonas fluorescens* | P*_ecf20_*__0992_ | *Pseudomonas fluorescens* |
|  | ECF42_4454 | *Xanthomonas campestris* | P*_ecf42_up406_*_2_ | *Methylibium petroleiphilum* |
|  | ECF41_0491 | *Caulobacter crescentus* | P*_ecf41_*__up1141_ | *Pseudomonas fluorescens* |
|  | ECF34_1384 | *Streptomyces coelicolor* | P*_ecf18_up1700_* | *Acidiphilium cryptum* |
|  | ECF28_1088 | *Shewanella frigidimarina* | P*_ecf19_up1315_* | *Streptomyces coelicolor* |
|  | ECF22_4450 | *Xanthomonas campestris* | P*_ecf22_up1147_* | *Xanthomonas axonopodis* |
|  | ECF16_3622 | *Pseudomonas entomophila* | P*_ecf16_3622_* | *Pseudomonas entomophila* |
|  | ECF32_1122 | *Erwinia amylovora* | P*_ecf32_1122_* | *Erwinia amylovora* |
|  | ECF17_1691 | *Mycobacterium tuberculosis* | P*_ecf17_up1691_* | *Mycobacterium tuberculosis* |
|  | ECF15_0436 | *Rhodobacter sphaeroides* | P*_ecf15_*__up436_ | *Rhodobacter sphaeroides* |
|  | ECF37_2513 | *Burkholderia thailandensis* | P*_ecf39_up1413_* | *Kineococcus radiotolerans* |
|  | ECF11_0987 | *Vibrio parahaemolyticus* | P*_ecf11_3726_* | *Pseudomonas syringae* |
|  | ECF03_1198 | *Bacteroides thetaiotaomicron* | P*_ecf03_up1198_* | *Bacteroides thetaiotaomicron* |
|  | ECF02_2817 | *Escherichia coli* | P*_ecf02_2817_* | *Escherichia coli* |
|  | ECF38_1322 | *Streptomyces coelicolor* | P*_ecf38_up1322_* | *Streptomyces coelicolor* |
|  | ECF33_0423 | *Rhodopseudomonas palustris* | P*_ecf33_375_* | *Bradyrhizobium japonicum* |
|  | ECF14_1324 | *Streptomyces coelicolor* | P*_ecf12_up807_* | *Anaeromyxobacter dehalogenans* |
|  | ECF26_4464 | *Xanthomonas oryzae* | P*_ecf26_up601_* | *Burkholderia sp.* |
|  | ECF31_0034 | *Bacillus subtilis* | P*_ecf31_34_* | *Bacillus subtilis* |
|  | ECF27_4265 | *Streptomyces coelicolor* | P*_ecf25_up4311_* | *Synechococcus sp.* |

color legend:

|  | Proteobacteria |
| --- | --- |
|  | Actinobacteria |
|  | Bacteroidetes |
|  | Firmicutes |

The ECF switches represent a highly orthogonal core set of regulators that have been implemented in *E. coli* in frame of a comprehensive pilot study [8]. A few promoters were modified by replacing the -35 to -60 region with an AT rich UP-element as indicated in the name by the prefix “up“.

**Table S8.** Classification of ECFs. Phylogenetic distribution of ECFs mentioned in this study according to the initial classification [13] and the recent classification [12].

|  | ECF name used in this study | Phylogenetic group  Staron et al. [13] | Phylogenetic group  Casas-Pastor et al. [12] | |
| --- | --- | --- | --- | --- |
|  |  |  | group | subgroup |
| Heterologous ECFs | ECF20_0992 | 20 | 290 | s1 |
|  | ECF42_4454^+^ | 42 | 42 | s1 |
|  | ECF42_4062* | 42 | 42 | s1 |
|  | ECF41_0491^+^ | 41 | 41 | s6 |
|  | ECF41_1141* | 41 | 41 | s16 |
|  | ECF34_1384^+^ | 34 | 19 | s4 |
|  | ECF18_1700* | 18 | ungrouped | - |
|  | ECF28_1088^+^ | 28 | 28 | s2 |
|  | ECF19_1315* | 19 | 19 | s1 |
|  | ECF22_4450^+^ | 22 | 22 | s3 |
|  | ECF22_1147* | 22 | 22 | s3 |
|  | ECF16_3622 | 16 | 16 | s1 |
|  | ECF32_1122 | 32 | 32 | s4 |
|  | ECF17_1691 | 17 | 17 | S4 |
|  | ECF15_0436 | 15 | 15 | s1 |
|  | ECF37_2513^+^ | 37 | 37 | s1 |
|  | ECF39_1413* | 39 | 39 | - |
|  | ECF11_0987^+^ | 11 | 11 | s5 |
|  | ECF11_3726* | 11 | 11 | s4 |
|  | ECF03_1198 | 03 | 03 | s8 |
|  | ECF02_2817 | 02 | 02 | s1 |
|  | ECF38_1322 | 38 | 38 | s1 |
|  | ECF33_0423^+^ | 33 | 33 | s1 |
|  | ECF33_0375* | 33 | 33 | s1 |
|  | ECF14_1324^+^ | 14 | 14 | s4 |
|  | ECF12_0807* | 12 | 12 | s5 |
|  | ECF26_4464^+^ | 26 | 26 | s4 |
|  | ECF26_0601* | 26 | 26 | s5 |
|  | ECF31_0034 | 31 | 31 | s1 |
|  | ECF27_4265^+^ | 27 | 27 | s1 |
|  | ECF25_4311* | 25 | 25 | s1 |
| *S. meliloti* ECFs | RpoE1 | 26 | 26 | s6 |
|  | RpoE2 | 15 | 15 | S1 |
|  | RpoE3 | 26 | 26 | s29 |
|  | RpoE4 | 26 | 26 | s34 |
|  | RpoE5 | 15 | 15 | S3 |
|  | RpoE6 | 26 | 26 | s14 |
|  | RpoE7 | 16 | 16 | S3 |
|  | RpoE8 | 29 | 29 | S1 |
|  | RpoE9 | 41 | 41 | s6 |
|  | RpoE10 | 42 | 42 | s7 |
|  | FecI | ungrouped | 243 | - |

(*) Only the autoregulatory promoter was used in this study; (+) the ECF but not the autoregulatory promoter was tested. Instead, the autoregulatory promoter of another *ecf* gene was used. Green shading refers to components of active ECF switches in the background of the ECF/anti-σ-free *S. meliloti* strain*.* Red shading of phylogenetic groups indicates re-classification of ECFs.

**Table S10.** Overview of significantly regulated genes in the ECF/anti-σ-free *S. meliloti* strains, overexpressing *ecf02* or *ecf11.*

| **significantly regulated genes of the ecf02 overexpression strain** | | | | | |
| --- | --- | --- | --- | --- | --- |
| **regulated gene** | **log_2_(*fc*)** | **p-value** | **putative operon** | **encoded protein function** | **further information** |
| SMb21421 | 4,8 | 2,7*10^-81^ | *SMb21421-SMb21424* | ABC transporter, periplasmic solute-binding protein | operon encoding a putative ATP-binding cassette (ABC) uptake system induced by tagatose [72] |
| SMb21422 | 5,1 | 2,4*10^-63^ | *SMb21421-SMb21424* | ABC transporter, ATP-binding protein |  |
| SMb21423 | 4,6 | 2,3*10^-73^ | *SMb21421-SMb21424* | ABC sugar transporter, permease |  |
| SMb21424 | 4,5 | 5,0*10^-117^ | *SMb21421-SMb21424* | putative acyl esterase |  |
| SMb20343 | 3,0 | 1,1*10^-102^ | *SMb20343- SMb20342* | putative isoquinoline 1-oxidoreductase | operon expression induced by phosphate starvation [73]  *Smb20342* encoded protein has twin-arginine transport motif and plays a role in symbiosis [74] |
| SMb20342 | 2,7 | 4,8*10^-17^ | *SMb20343- SMb20342* | putative isoquinoline 1-oxidoreductase |  |
| SMb20094 | 2,7 | 2,8*10^-16^ | *-* | phospholipase D | *SMb20094* is regulated by RpoE2 [75] |
| SMc00763 | 2,3 | 5,0*10^-72^ | *-* | conserved hypothetical protein | - |
| *SMc04206 | 1,6 | 4,6*10^-8^ | *SMc04206- SMc04208* | putative hemolysin-type calcium-binding protein | - |
| SMc04207 | 2,2 | 2,2*10^-10^ | *SMc04206- SMc04208* | rhizobiocin secretion protein |  |
| *SMc04208 | 1,7 | 3,3*10^-5^ | *SMc04206- SMc04208* | rhizobiocin secretion protein RspE |  |

*Note that *SMc04206* and *SMc04208* are shortly below the applied threshold for significantly regulated genes, while expression of *SMc0407* is above. Since those three likely are arranged in one operon, *SMc04206* and *SMc04208* are included in the table.

**Table S12.** Growth factors of ECF/anti-σ deletion strains carrying one-step and two-step timers employing different experimental setups. Growth factors have been determined from exponentially growing cells up to seven hours after induction of *ecf* expression in the absence of IPTG and under fully induced conditions in the presence of 500 µM IPTG using the Python package croissance v1.2.0. Growth factors +/- standard deviation of four biological replicates are shown. Underlying data can be extracted from Table S11.

| **main characteristics of ECF circuit** | | | **number of pABC plasmids** | **growth factor   0 µM IPTG** | | | **growth factor 500 µM IPTG** | | |
| --- | --- | --- | --- | --- | --- | --- | --- | --- | --- |
| **input module** | **delay module** | **output module** |  |  |  |  |  |  |  |
| P*_lac_*_T5_ : *ecf20** | - | P*_ecf20_* : *luxCDABE* | 2 | 0,236 | ± | 0,006 | 0,219 | ± | 0,006 |
| P*_lac_*_T5_ : *rpoE4* | - | P*_ecf26_* : *luxCDABE* | 2 | 0,227 | ± | 0,012 | 0,293 | ± | 0,044 |
| P*_lac_*_T5_ : *ecf20** | P*_ecf20_* : *rpoE4* | P*_ecf26_* : *luxCDABE* | 3 | 0,231 | ± | 0,007 | 0,236 | ± | 0,005 |
| P*_lac_*_T5_ : *rpoE4* | P*_ecf26_* : *ecf20** | P*_ecf20_* : *luxCDABE* | 3 | 0,231 | ± | 0,003 | 0,231 | ± | 0,002 |
| P*_lac_*_T5_ : *ecf20** | P*_ecf20_* : *rpoE4* | P*_ecf26_* : *luxCDABE* | 1 | 0,227 | ± | 0,009 | 0,240 | ± | 0,014 |
| P*_lac_*_T5_ : *rpoE4* | P*_ecf26_* : *ecf20** | P*_ecf20_* : *luxCDABE* | 1 | 0,230 | ± | 0,005 | 0,234 | ± | 0,003 |

**
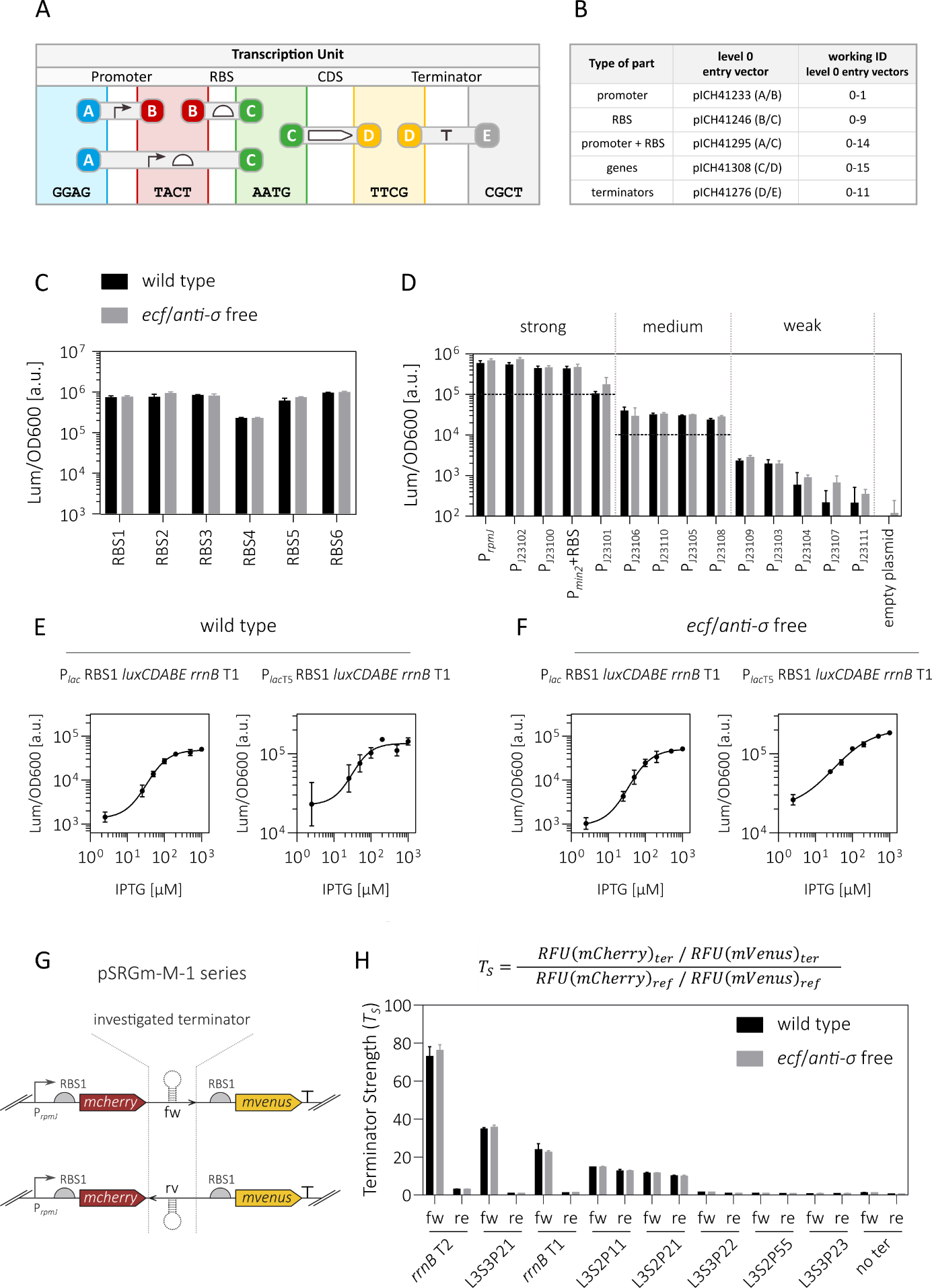
**

**Fig. S1.** MoClo standard of basic parts. (A) Type, position, and fusion sites of basic parts are detailed. Part specific fusion sites are shown and further indicated by capital letters and specific colours. Level 0 destination vectors carrying the standard elements were obtained from Weber et al. [25]. (B) Level 0 entry vectors [25] for specific parts are indicated. For simplicity, working IDs introduced by Pinto et al. [7] and Veccchione et al. [18] are used for level 0 vectors. (C) Strength of different RBS (labeled RBS1-6, detailed specifications in Table S3) were analyzed in Rm1021 and the ECF/anti-σ free strain. Transcription units composed of the constitutive *rpmJ* promoter, an RBS, the *lux* operon and the *rrnB* T1 terminator (from *E.* coli) were assembled in pABCa-1-1a-mob. Constructs were transferred in both strains. Luminescence units per unit of OD_600_ were determined 9 hours after cell synchronization. (D) Several constitutive promoters (detailed specifications in Table S3) were characterized in Rm1021 and the ECF/anti-σ free. Transcription units composed of one constitutive promoter, RBS1, the *lux* operon, and the *rrnB* T1 terminator were assembled in pABCa-1-1a-mob and transferred to both strains. Relative luminescence units correlating with promoter strength were determined as described in C. (E, F) Dose response characteristics of the IPTG inducible promoters P*_lac_* [34] and P*_lac_*_T5_ [60] fused to the *lux* operon were analyzed in Rm1021 and the ECF/anti-σ free strain 9 hours after the addition of IPTG at indicated concentrations. Luminescence units per unit of OD_600_ were determined. Transcription units were assembled according to the description in D. (G) Schematic representation of the experimental setups used to determine termination efficiencies of Rho-independent terminators in forward or in reverse direction. The terminators to be characterized were inserted between *mcherry* and *mvenus* genes in an operon carried by the multi-copy plasmid pSRGm-M-1 (Table S3). (H) Plasmids generated to measure termination efficiencies were transferred to Rm1021 and the ECF/anti-σ free strain. mCherry and mVenus fluorescence were determined 12 hours after cell synchronization. The formula shown was used to determine terminator strength (*Ts*) according to [38]. Underlying data from C-D can be extracted from Table S11.


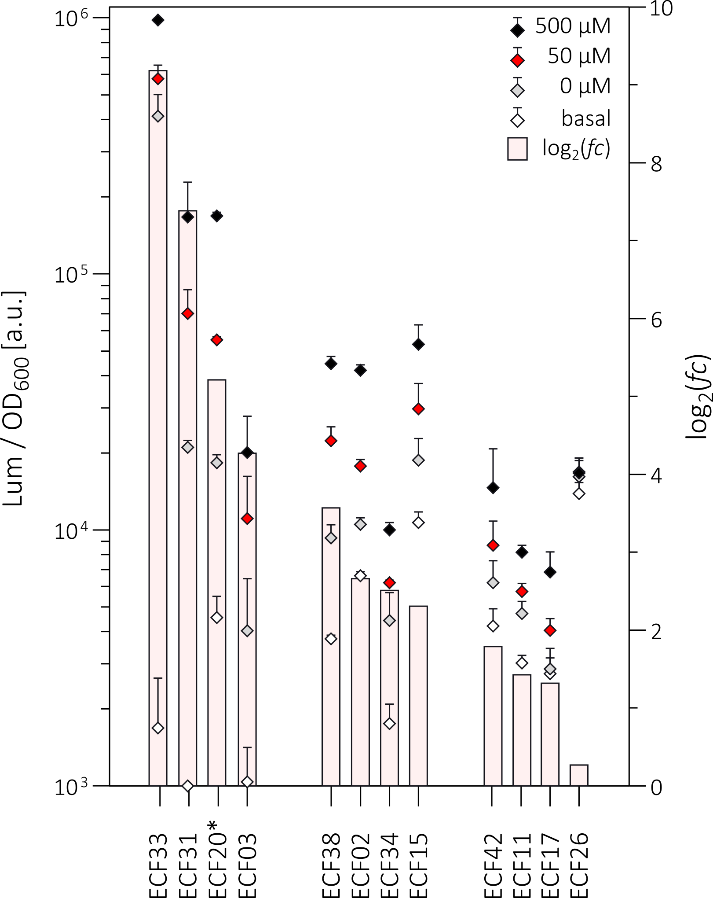


**Fig. S2.** Performance of active heterologous ECF switches in the *S. meliloti* Rm1021 wild type*.* The experimental setup depicted in Fig. 1A was applied. Different IPTG concentrations (0, 50 and 500 µM) were used to induce *ecf* expression. Promoter activities were normalized to yield luminescence units per unit of OD_600_ (left y-axis)_._ To determine basal *ecf* promoter activities (basal), luminescence was assayed in strains carrying an empty pABCb mob plasmid without any heterologous *ecf* gene. Each dot represents the mean response of three biological replicates 6 hours after addition of IPTG. Error bars represent standard deviation. Rectangular bars indicate the average log2 fold induction of promoter activity (right y-axis) in the presence of 500 µM IPTG compared to the basal promoter activity in the absence of any heterolougs *ecf*. Raw data are shown in Table S11**.** An asterisk (*) is indicative for an ECF with a 6xHis tag fusion at its N-terminus (Table S3).


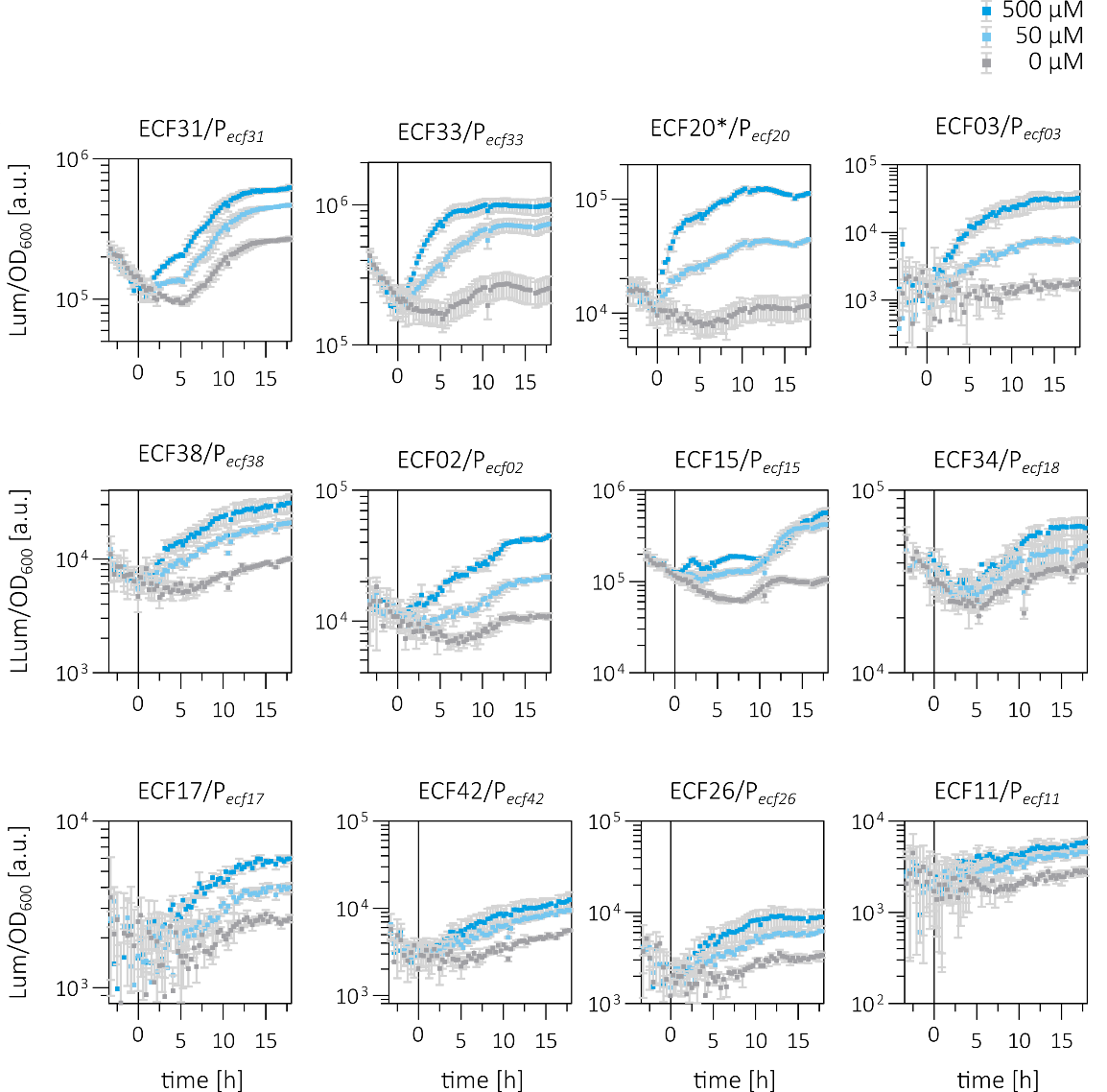


**Fig. S3.** The dynamic response of single-copy ECF switches in *S. meliloti.* Two single-copy pABC plasmids carry either the transcriptional unit for IPTG-dependent *ecf* expression (pABCb mob series) or the cognate *ecf* promoter driving expression of a *luxCDABE* reporter (pABCa mob series). Plasmids with cognate *ecf/*P*_ecf_* pairs were transferred to the ECF/anti-σ-free *S. meliloti* strain [48]. Expression of the *ecf* gene was induced at time point t=0 with different IPTG concentrations: 0 (grey colored line), 5, 50, 500 µM IPTG (shades of blue colored lines). P*_ecf_* activity (shown in relative luminescence units normalized by OD_600_) was determined during bacterial growth for three biological replicates. Error bars represent standard deviation. Note that the range of the Y-axis varies between switches. Raw data are given in Table S11. An asterisk (*) is indicative for an ECF with a 6xHis tag fusion at its N-terminus (Table S3).


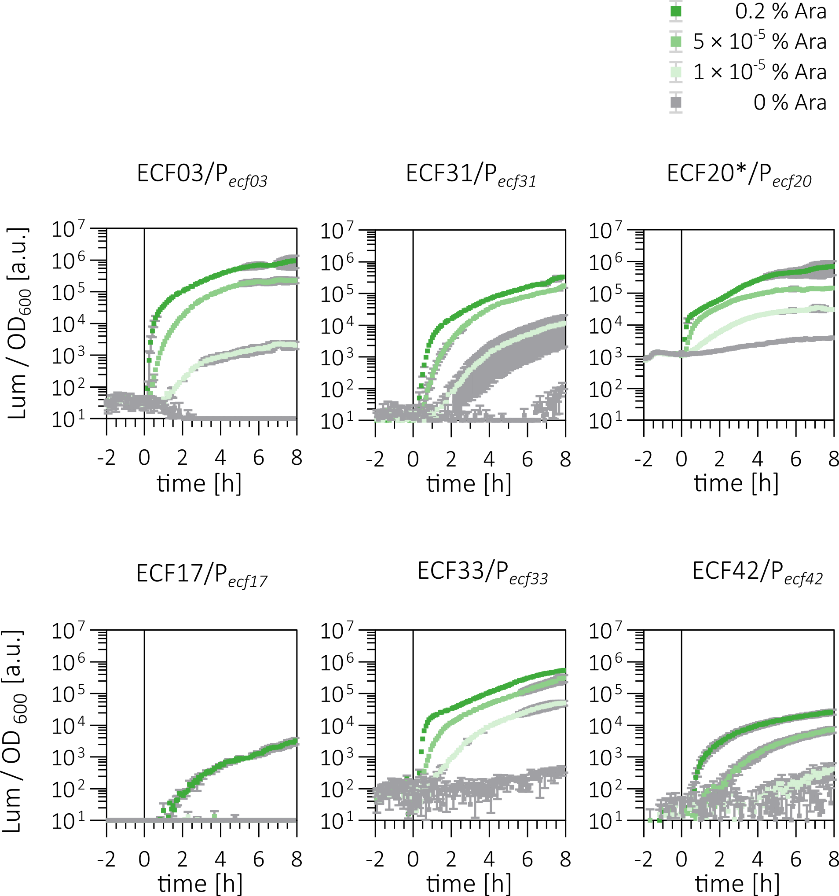


**Fig. S4.** The dynamic response of relevant single copy *ecf* switches in *E. coli* that have not been analyzed by Pinto et al. [7] before. P*_BAD_ ecf* transcription unit and cognate P*_ecf_* transcription units were cloned into the CriMoClo vector pSV004. Plasmids carrying *ecf* circuits were integrated into the chromosome of *E. coli* strain SV001 at the HK022 attachment site. Expression of the *ecf* gene was induced at t_0_ with 0, 10^-5^, 5x 10^-5^, 2x 10^-1^ % of arabinose. P*_ecf_* activity (shown in relative luminescence units normalized by OD_600_) was determined every 5 min during bacterial growth for three biological replicates. Error bars represent standard deviation. Raw data are shown in Table S11. . An asterisk (*) is indicative for an ECF with a 6xHis tag fusion at its N-terminus (Table S3).


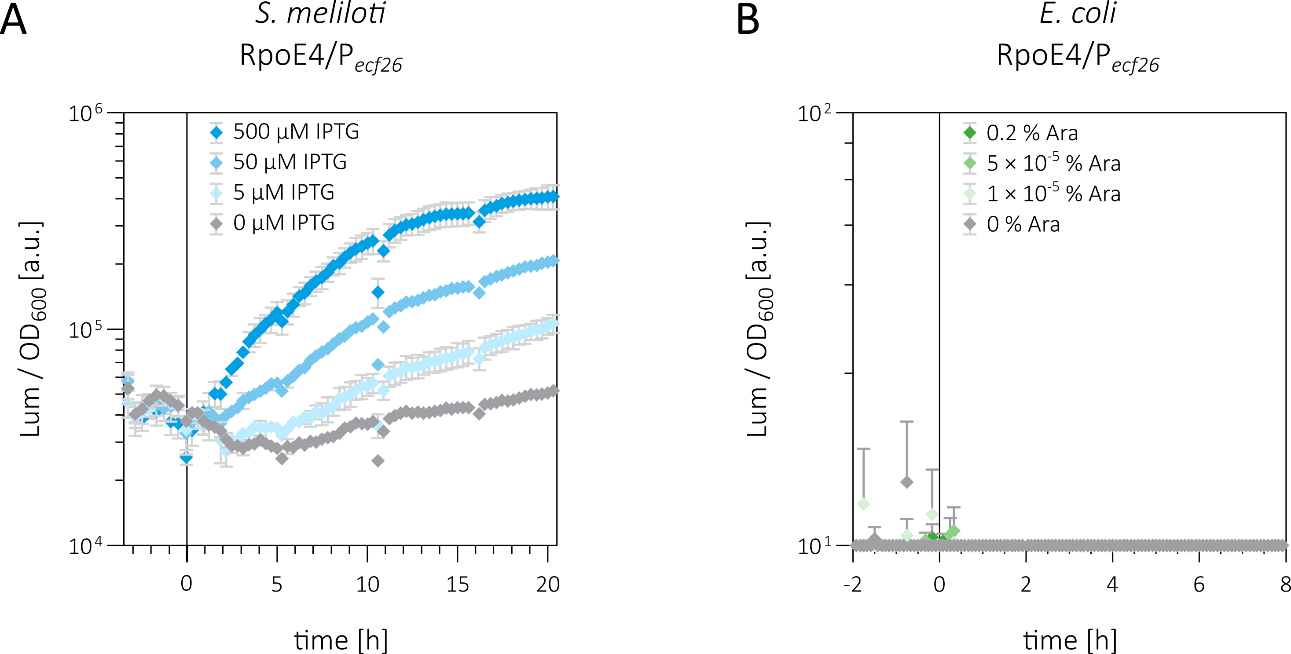


**Fig. S5.** Dynamic response of the ECF switch composed of RpoE4 and P*_ecf26_* in *S. meliloti* and *E. coli*. The ECF circuit was carried by single-copy pABCa-/b-mob derivatives in the ECF/anti-σ-free S. meliloti strain or it was integrated into the chromosome of the E. coli strain SV01. Panels (a) and (b) show the response of luciferase activity (relative luminescence normalized by the OD600) for three biological replicates. Expression of the ecf gene was induced with 0, 5, 50, 500 µM IPTG (*S. meliloti*) or with 0, 10-5, 5x 10-5, 2x 10-1 % of arabinose (*E. coli*) at t = 0 h (black solid line). The basal luciferase activity (absence of any inducer) is shown as grey solid line. Colored lines in shades of blue show the specific response after induction of ecf expression with increasing inducer concentrations. Error bars represent standard deviation. Raw data are shown in Table S11.

**
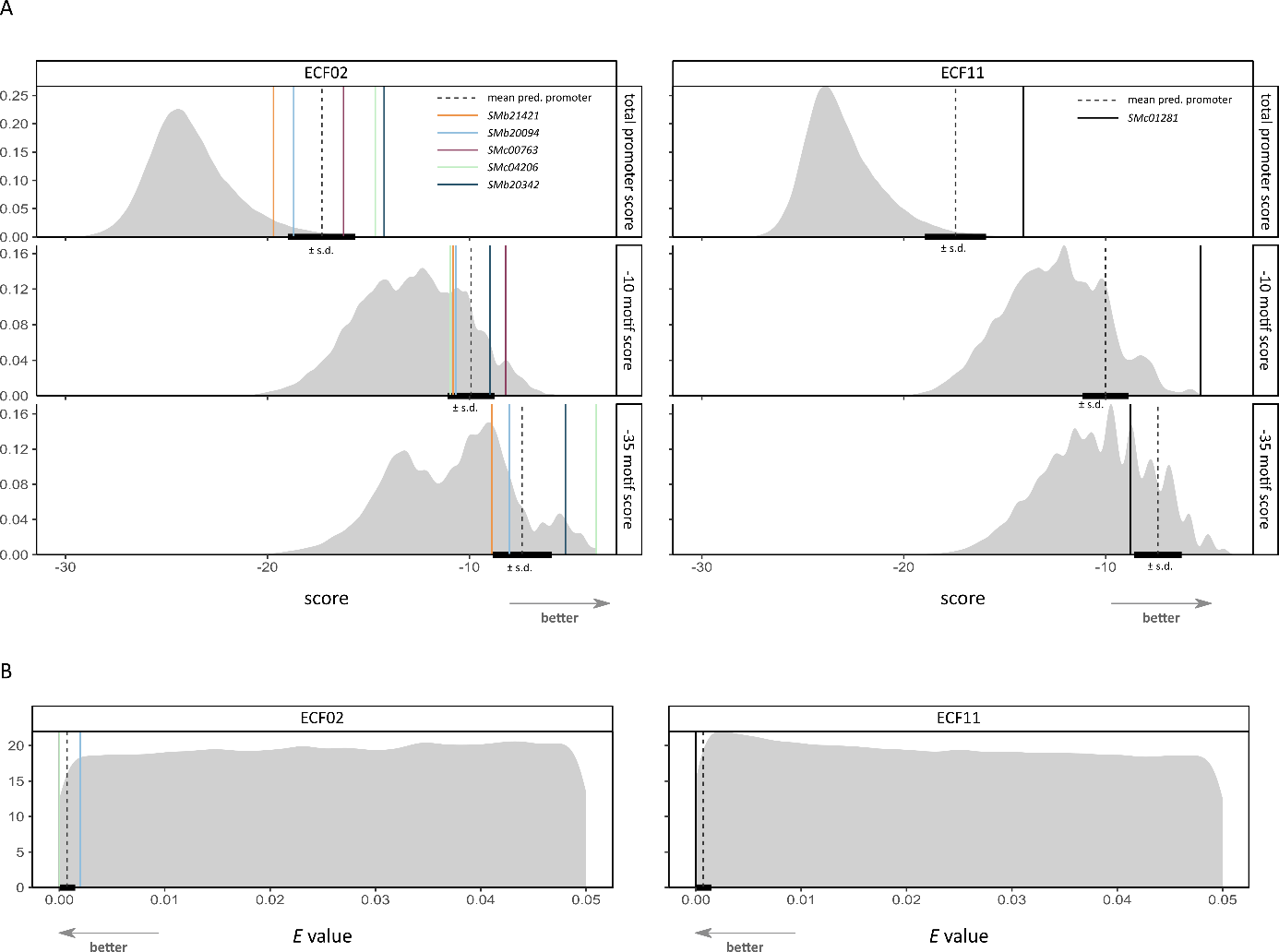
**

**Fig. S6.** Distribution of total promoter scores and *E*-values from promoters computationally predicted to be associated with off-target gene transcription in *ecf02* and *ecf11* overexpression strains. (A) Distribution of log promoter scores obtained by searching the -35 and -10 promoter PSSMs from ECF02 and ECF11 (including a linker of 15 to 17 bp) against the genome of Rm1021 (grey). Distributions of -35 and -10 motif scores obtained from the combined search of promoter PSSMs are also shown. Vertical (colored) lines correspond to total promoter, or -35 and -10 motif scores associated with upregulated genes in the RNAseq data. For simplicity, the first gene of regulated operons is used as label. Genes computationally predicted to be ECF-regulated are represented by dashed vertical lines, indicating mean promoter or -35 and -10 motif scores. Standard deviation can be seen as thick horizontal line. (B) *E*-values associated with computationally predicted *ecf* promoter scores from A. Note that vertical lines corresponding to genes *SMb21421*, *SMc00763* and *SMb20342* are hidden between the green line representing *SMc04206*.


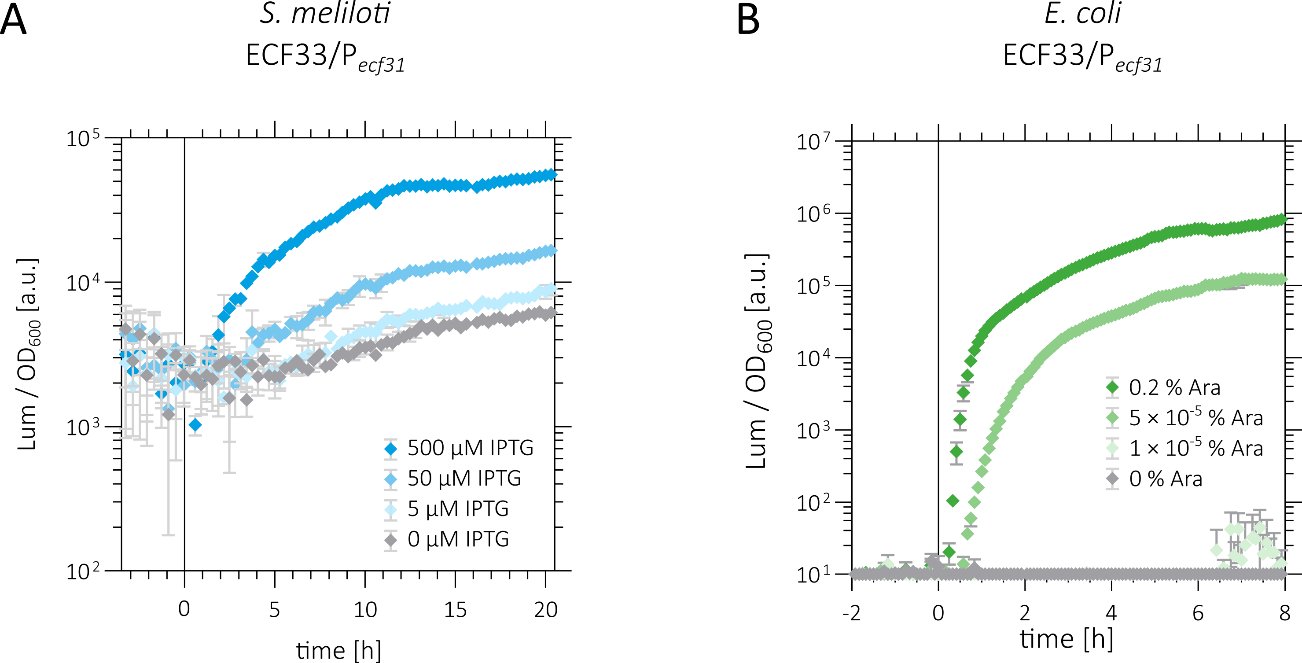


**Fig. S7.** Dynamic response of the ECF switch composed of ECF33 and P*_ecf31_* in *S. meliloti* and *E. coli*. The ECF circuit was carried by single-copy pABCa-/b-mob derivatives in the ECF/anti-σ-free *S. meliloti* strain or it was integrated into the chromosome of the *E. coli* strain SV01. Panels (a) and (b) show the response of luciferase activity (relative luminescence normalized by the OD_600_) for three biological replicates. Expression of the *ecf* gene was induced with 0, 5, 50, 500 µM IPTG (*S. meliloti*) or with 0, 10^-5^, 5x 10^-5^, 2x 10^-1^ % of arabinose (*E. coli*) at t = 0 h (black solid line). The basal luciferase activity (absence of any inducer) is shown as grey solid line. Colored lines in shades of blue show the specific response after induction of *ecf* expression with increasing inducer concentrations. Error bars represent standard deviation. Raw data are shown in Table S11.


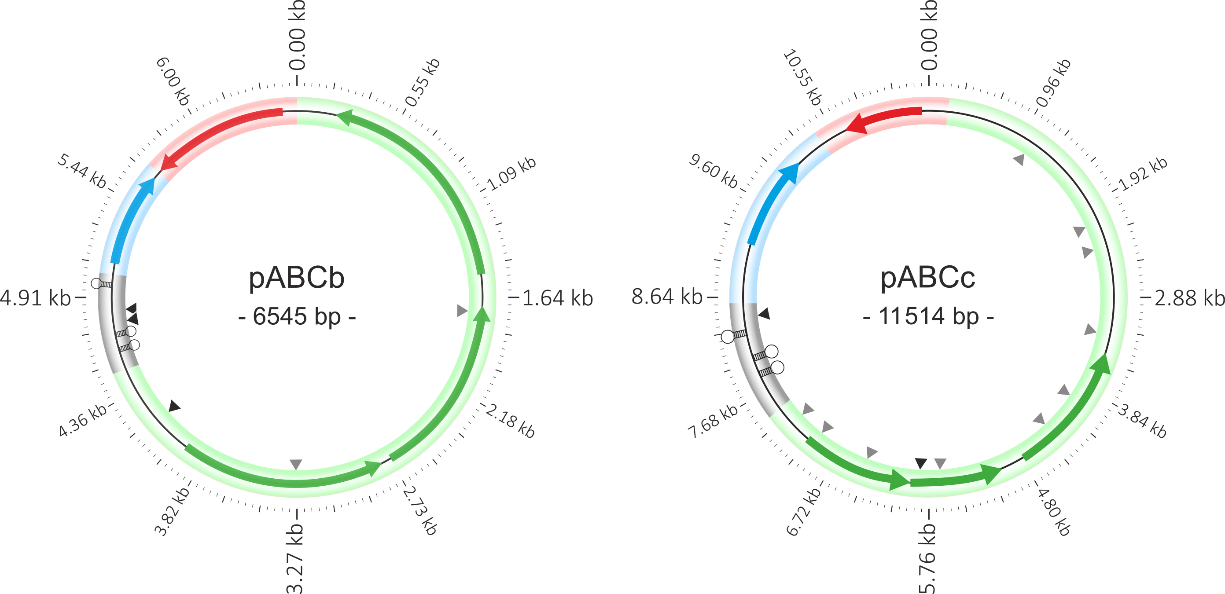


**Fig. S8.** Schematic representation of pABCb and pABCc plasmids [31] and the position and number of BsaI and BpiI sites. Arrows represent open reading frames (ORFs). Colors refer to specific module parts: oriVSm (green), oriVEc (blue), AR (red), synterMCS (grey). BpiI and BsaI sites are highlighted by grey and black triangles, respectively. Most sites are present in the oriVSm module of the plasmids, namely 3/5 and 11/12 in case of pABCb and pABCc, respectively. The others were found in the synterMCS region.


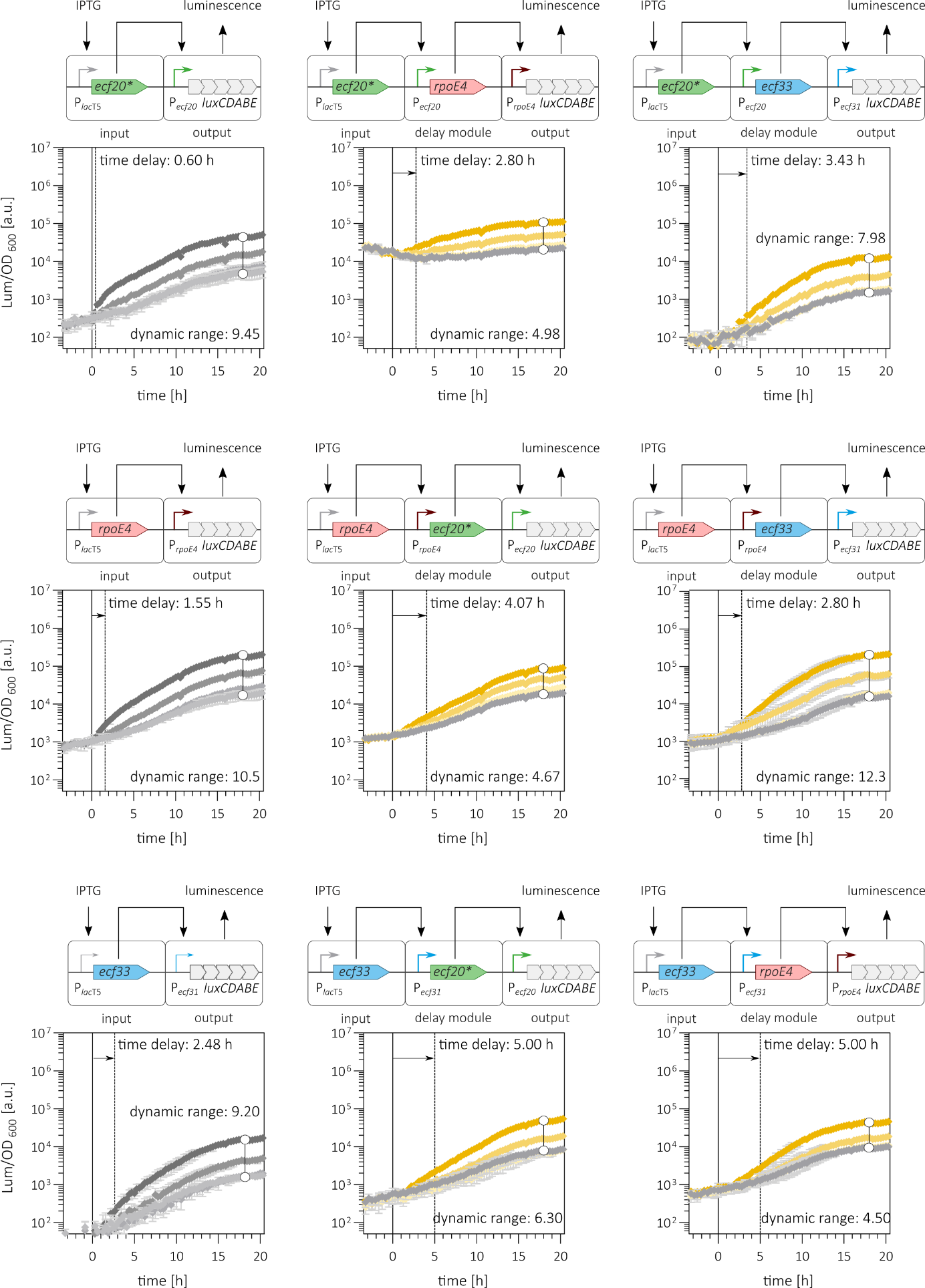


**Fig. S9.** Performance of 1-step and 2-step delay circuits in *S. meliloti*. The principle designs of all circuits are shown as cartoons. Detailed information on transcription units can be extracted from Table S3. Plasmids carrying the input module (pABCb mob based) the delay module (pABCc mob based) and the output module (pABCa mob based) were transferred by conjugation into the ECF/anti-σ-free strain. In case of the 1-step timers, an empty pABCc mob plasmid without delay module was used. Panels show the response of luciferase activity (relative luminescence normalized by the OD_600_). Expression of the first *ecf* gene of each circuit was induced with 0, 5, 50 and 500 µM IPTG at t = 0 h (black solid line). The basal luciferase activity (absence of any inducer) is shown as grey solid line. Colored lines in shades of yellow (2-step timers) and black (1-step timers) show the specific response after induction of *ecf* expression with increasing inducer concentrations. Error bars represent standard deviation including measurements of at least three biological replicates. Dashed lines indicate the time delay after addition of maximum inducer concentrations until average luminescence exceeded basal luminescence by at least 2-fold. The maximum dynamic output range (dr) was determined 18 hours after inducer addition. An asterisk (*) is indicative for an *ecf* gene that has been translationally fused with a 6xHis tag encoding sequence (Table S3).


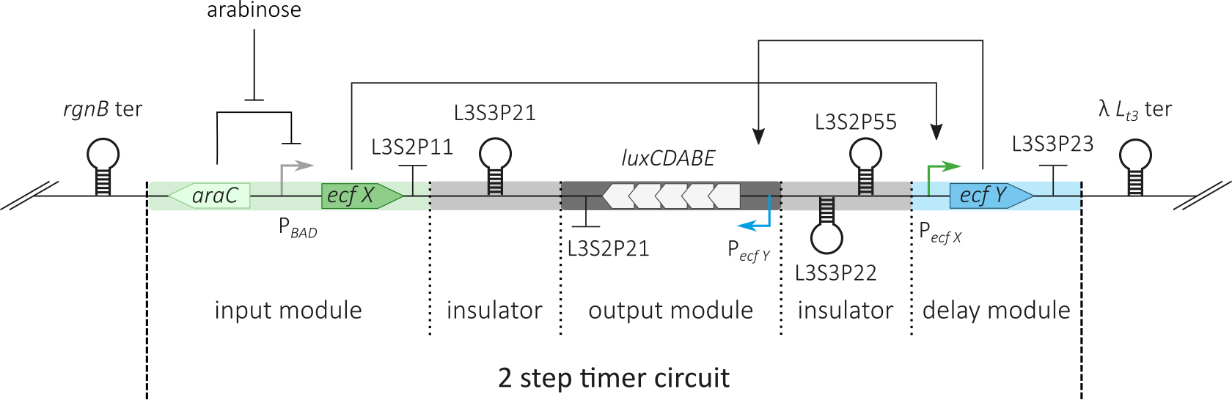


**Fig. S10.** Scheme representing the arrangement of transcription units and transcriptional insulators of chromosomally integrated 2-step timers in *E. coli* adopted from Pinto et al. 2018. Terminators L3S1P11, L3S3P21, L3S2P21, L3S2P55 and L3S3P23 were previously characterized [61]. Thick, dashed lines confine the assembled 2-step timer circuit. Dotted lines define DNA-units that were assembled in the pSV006 vector backbone. *RgnB* ter and λ *L*_t3_ are terminators that were already present in the vector backbone.


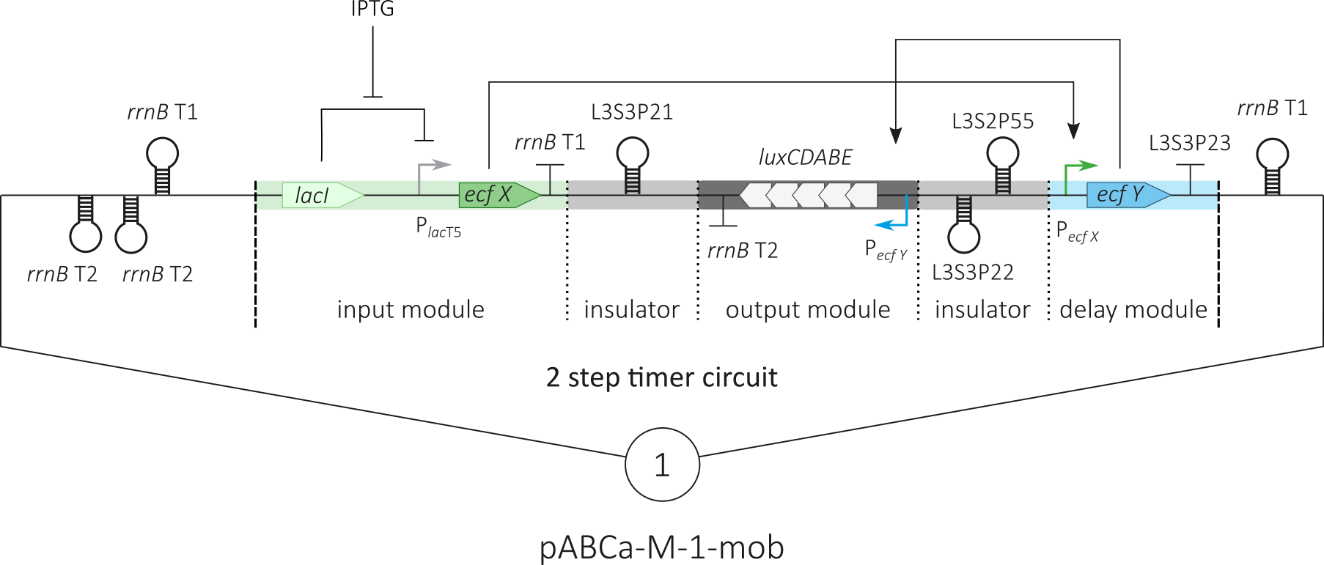


**Fig. S11.** Scheme representing the arrangement of transcription units and transcriptional insulators of 2-step timers carried by pABCa-M-1-mob in *S. meliloti*. Thick, dashed lines confine the assembled 2-step timer circuit. Dotted lines define DNA-units that were assembled in the pABCa-M-1-mob vector backbone. Terminators of insulating parts were adopted from Pinto et al. [7]. Note that terminators flanking the 2-step timer circuit are part of the vector backbone. Employed terminators have been characterized in *S. meliloti* (Fig. S1).
